# SARS-CoV-2 nonspike structural proteins hijack mucosa epithelial cell fate

**DOI:** 10.1038/s41419-026-08611-6

**Published:** 2026-03-23

**Authors:** Yan Gao, Lucas Lacerda Souza, Hong Soon Kang, Zehan Li, Juan Carlos Hernandez-Guerrero, Fábio Abreu Alves, Wei Zhang, Vikram Sharma, Sally Hanks, Jinhua Yu, Christopher Tredwin, Anton M. Jetten, Ciro Dantas Soares, Bing Hu

**Affiliations:** 1 School of Stomatology, Southern Medical University, Guangzhou, Guangdong China; 2 Guangdong Institute of Dental and Craniofacial Research, Guangzhou, Guangdong China; 3 Peninsula Dental School, Faculty of Health, University of Plymouth, Plymouth, United Kingdom; 4 Department of Oral Diagnosis, Piracicaba Dental School/State University of Campinas, Piracicaba, Brazil; 5 Cell Biology Group, Immunity, Inflammation and Disease Laboratory, National Institute of Environmental Health Sciences, National Institutes of Health, Research Triangle Park, Durham, NC USA; 6 Jiangsu Key Laboratory of Oral Diseases, Nanjing Medical University and Department of Stomatology, Nanjing Medical University, Nanjing, China; 7 Department of Endodontics, Affiliated Hospital of Stomatology, Nanjing Medical University, Nanjing, China; 8 Department of Immunology, Universidad Nacional Autónoma de México, Mexico City, Mexico; 9 Department of Stomatology, School of Dentistry, University of São Paulo, São Paulo, Brazil; 10 Stomatology Department, A.C. Camargo Cancer Center, São Paulo, Brazil; 11 Department of Pathology, Affiliated Hospital of Stomatology, Nanjing Medical University, Nanjing, China; 12 School of Biomedical Sciences, Faculty of Health, University of Plymouth, Plymouth, United Kingdom; 13 Institute of Dentistry, Faculty of Medicine and Dentistry, Queen Mary University of London (QMUL), Turner Street, London, United Kingdom; 14 Department of Pathology, Getúlio Sales Diagnósticos, Natal, Rio Grande do Norte Brazil

**Keywords:** Cell death, Viral infection, Transcriptional regulatory elements

## Abstract

COVID-19 patients readily present with severe epithelial damage, such as tissue ulceration and erosion, along with disrupted tissue repair, in multiple organs. The mucous membranes of the lung alveoli [1, 2], gastrointestinal tract [3, 4], nasal [5] and oral cavity [6, 7] are the primary targets of the SARS-CoV-2 virus. The infected epithelium triggers a dysregulated immune response that further damages tissues and organs [8–10]. Increasing evidence suggests that the SARS-CoV-2 virus can cause direct damage to epithelial cells and fibroblasts [11–13]. Here, we report that the mucosa epithelia of COVID-19 patients can undergo cellular dedifferentiation before any pathological features are observed. SARS-CoV-2 nonspike structural proteins, particularly the Envelope protein, can rapidly induce epithelial cell dedifferentiation, micronuclei formation, cell cycle arrest at the G1 phase and apoptosis. The protein can also severely affect the progenitor cell stratification program. Mechanistically, we identified a unique molecule, calponin 2 (CNN2), as a downstream effector of nonspike structural proteins. Moreover, CNN2 levels were elevated in the epithelia of COVID-19 patients. Downregulating CNN2 could inhibit epithelial cell apoptosis and promote cell differentiation. CNN2 expression is negatively regulated by GLIS2, a transcription factor associated with the disruption of ciliary dynamics in epithelial cells. Therefore, we propose that SARS-CoV-2 damages mucosal epithelium integrity via a novel “double hijack” mechanism: inducing dedifferentiation and disrupting stratification and suggest a new therapeutic target: CNN2 for COVID-19 treatment.

## Human mucosal epithelial cell dedifferentiation is a common feature of early SARS-CoV-2 infection

Epithelial tissues line the internal cavities of the human body and act as the first line of defense against external pathogens and irritants. The SARS-CoV-2 virus can enter most epithelial tissues, trigger severe inflammation, and cause necroptosis and apoptosis. In the airway and pulmonary epithelium, it destroys the air‒blood barrier. In the kidney, it induces tubular injury [13, 14]. In the oral cavity, throat and gastrointestinal tract, it causes severe ulceration [7, 15, 16].

**Fig. 1:**
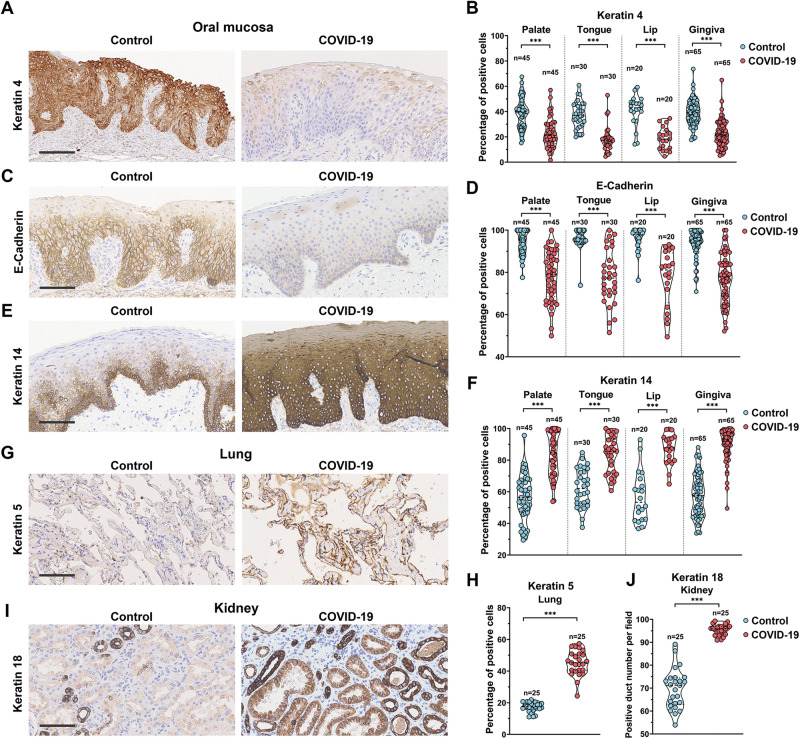
Epithelial cell dedifferentiation in the COVID-19 patient oral mucosa, lung and kidney epithelium. **A**, **C**, **E**, **G**, **I** Representative immunohistochemistry analysis of the corresponding markers on COVID-19 patient samples. Additional images can be found in Supplementary Figs. S2, S3. **B**, **D**, **F**, **H**, **J** Quantitative analysis of positive cells was performed on random 5 fields per field were analyzed for each patient’s samples. n total number of the image fields quantified. For statistical test results, ***p < 0.001, center values represent mean and error bars represent s.d. Bars: 100 μm.

Epithelial cell dedifferentiation is a rare biological event that mainly occurs during tissue repair to repair damaged epithelial tissues in the airway, kidney, and intestine [17–19]. Epithelial cell dedifferentiation has been observed in SARS-CoV-2-infected human bronchial epithelial cells in vitro and hamster airway epithelial tissues in vivo [20], and also in cultured human kidney organoids [13]. Differentiated cells have distinctive markers compared with undifferentiated cells, and the markers are tissue dependent. We first evaluated the occurrence of epithelial cell dedifferentiation in COVID-19 patient tissues with a focus on the oral mucosa, and the lung epithelium and kidney epithelium were used as comparisons (Supplementary Fig. S1A). Using immunohistochemistry analysis, we demonstrated that ACE2 and TMPRSS2, the two receptors for SARS-CoV-2, are widely expressed in the human oral mucosa, particularly in the suprabasal layers, which contain mainly postmitotic differentiating cells (Supplementary Fig. S1A, B). Our results revealed that in areas with no obvious inflammation, i.e., where the epithelium was morphologically normal but the SARS-CoV-2 virus was already present (Supplementary Fig. S1C), in the oral mucosa of COVID-19 patients (32 patients, vs. 32 healthy controls, 5 fields for each sample) the expression of the cell differentiation markers Keratin 4 (Fig. 1A, B and Supplementary Fig. S2A) and E-Cadherin (Fig. 1C, D and Supplementary Fig. S2B) were decreased, while the undifferentiation marker Keratin 14 was increased (Fig. 1E, F and Supplementary Fig. S2C). Similarly, in the lung and kidney epithelia of COVID-19 patients (5 patients vs. healthy controls, 5 fields for each sample), the expression of the undifferentiated markers Keratin 5 (Fig. 1G, H and Supplementary Fig. S3A) and Keratin 18 (Fig. 1I, J and Supplementary Fig. S3B) was also significantly elevated. Our results confirmed for the first time that epithelial cell dedifferentiation is a common clinical pathological feature of early SARS-CoV-2 infection that is likely not due to inflammation.

## SARS-CoV-2 nonspike structural proteins can directly induce epithelial cell dedifferentiation

**Fig. 2:**
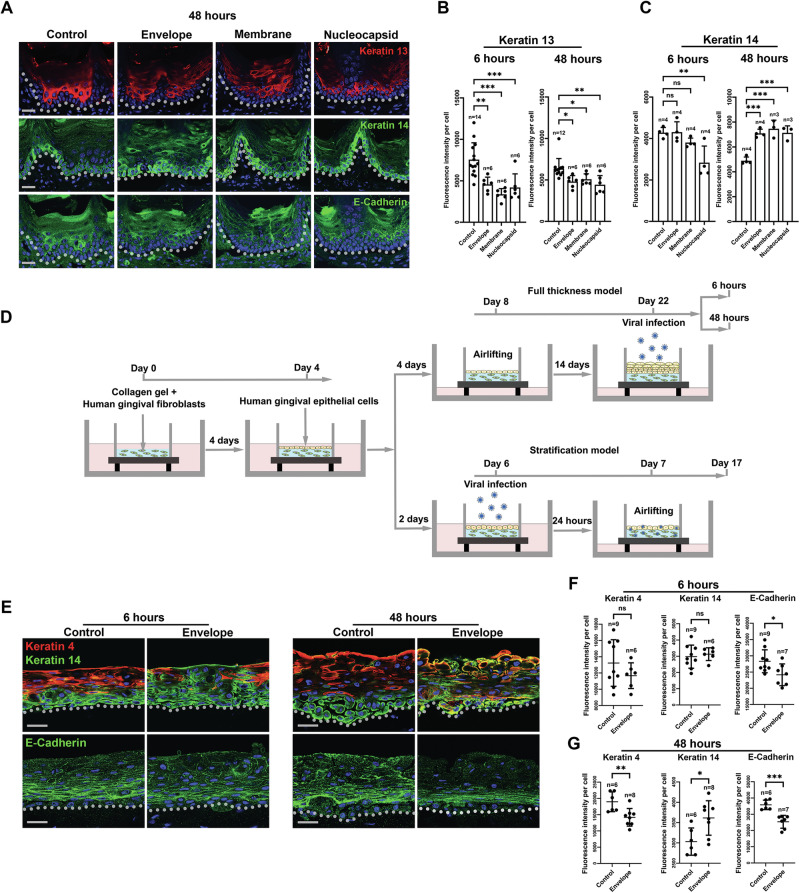
SARS-CoV-2 nonspike structural proteins can induce mouse tongue epithelial cell dedifferentiation. **A** Representative images of immunofluorescent analysis of the corresponding indicated markers for samples collected after 48 h infection. For 6 h samples please see Supplementary Fig. S5A. **B**, **C** Quantitative analysis of Keratin 13 and Keratin 14 signal intensity per cell in the tongue epithelium. For E-Cadherin quantification results please see Supplementary Fig. S5B. **D** Illustration of the 3D human oral mucosa equivalent systems: full thickness and stratification models. For the normal full-thickness model please see Supplementary Fig. S6A, B. **E**–**G** Representative immunofluorescence analysis images and quantification of the corresponding markers and quantification in the 3D oral mucosa full-thickness infection model. Dotted lines indicate epithelial-mesenchymal junctions. *n* total number of the image fields quantified. For statistical test results, center values represent mean and error bars represent s.d. ns no significance (*p* ≥ 0.05); *p < 0.05; **p < 0.01; ***p < 0.001. Bars: 20 μm.

SARS-CoV-2 contains four structural proteins, namely, the spike (S), envelope (E), membrane (M), and nucleocapsid (N) proteins, and sixteen nonstructural proteins (nsp1–16) [21]. The S protein, which is responsible for attaching the virus to host cells, has continuously mutated over time. In contrast, E, M and N protein mutations rarely occur [22]. In this study, we focused on the E, M and N proteins. To evaluate their roles in epithelial cell dedifferentiation, we first established a mouse tongue organ culture system (Supplementary Fig. S4A). Like the human oral mucosa, the mouse tongue mucosal epithelium also coexpresses ACE2 and TMPRSS2 (Supplementary Fig. S4B). We infected the mouse tongues with a lentiviral pseudovirus carrying genes encoding either the E, M, or N protein [23], for 6 h to simulate acute infection and 48 h to represent chronic infection [11]. The infection efficiency was verified with immunostaining of specific antibodies targeting the E, M, or N protein, which showed full-thickness epithelial penetration of the viral proteins were already observable after 6 h and highly elevated after 48 h (Supplementary Fig. S4C, D). Our results also revealed that all three proteins could quickly decrease Keratin 13 expression (Fig. 2A, B and Supplementary Fig. S5A) and increase Keratin 14 expression at both time points (Fig. 2A, C and Supplementary Fig. S5A). However, E-cadherin was significantly downregulated only by the E protein after 48 h (Fig. 2A and Supplementary Fig. S5A, B). To validate the above findings, we established human three-dimensional human oral mucosa equivalents to investigate the functions of the E protein (Fig. 2D and Supplementary Fig. S6A, B). The efficiency of viral infection was validated with Strep Tag II staining, which all the viral vectors used in the study carried. The results showed that both in 6 and 48 h, the virus could reach the full layers of the epidermis ubiquitously (Supplementary Fig. S6C, D). The results also showed indeed revealed that the E protein could decrease Keratin 4 and E-Cadherin expression and increase Keratin 14 expression (Fig. 2E–G). Moreover, we also observed an increased proportion of apoptotic cells in suprabasal layer cells (Supplementary Fig. S6E–G).

**Fig. 3:**
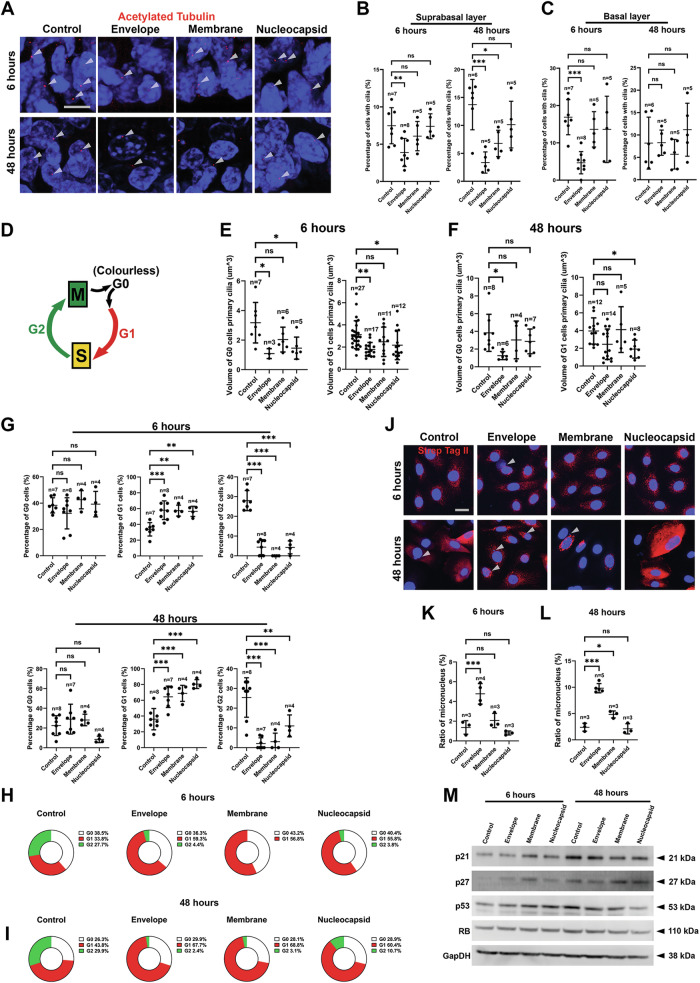
SARS-CoV-2 non-spike structural proteins cause cellular and cell cycle changes. **A** Representative images showing cilia in the mouse tongue organ cultures under indicated different conditions. White arrowheads indicate cilia. **B**, **C** Quantification of cilia number in the corresponding conditions that also showed in (**A**). *n* total number of fields quantified. **D** Simplified illustrative drawing of the ki67 FUCCI system used in the current system with color index (note cells at G0 phase have no color). **E**, **F** Cilia size analysis for infected cells (identified as positive cells stained by individual-specific antibodies as shown in Supplementary Fig. S10) in G0 and G1 phases under each condition. Additional analysis can be found in Supplementary Figs. S11A, B. *n* the number of cilia included in the quantification. **G**–**I** Analysis of the percentage of cells at different cycle phases under different infection conditions. *n* number of the fields quantified. **J**–**L** Micronuclei analysis in the cultured HGEPp cells and quantification with counter staining of Strep Tag II for identifying positively infected cells. White arrowheads indicate micronuclei. *n* number of the image fields quantified. **M** Western blotting analysis of the corresponding markers under indicated infection conditions and time points. For quantification, please see Supplementary Fig. S11C. For statistical test results, center values represent mean and error bars represent s.d. ns no significance (*p* ≥ 0.05); *p < 0.05; **p < 0.01; ***p < 0.001. Bars: 10 μm.

Cilia resorption can be a concurrent event with epithelial cell dedifferentiation [24]. Recent in vitro modeling studies have suggested that ciliary dynamics in lung epithelial cells can be disrupted in SARS-CoV-2-infected tissues and that cell dedifferentiation can be induced [19], although direct clinical evidence has not yet been found. We next evaluated the status of cilia in epithelial cells in mouse tongue organ cultures. We found that the number of ciliated cells significantly decreased, particularly in the E group, but not in the M and N groups (Fig. 3A–C). To validate the findings in mouse tongue organ cultures, we next evaluated the effects of the E, M and N proteins in cultured human gingival epithelial cells. We first validated the infection efficiency using immunostaining (Supplementary Fig. S7A, B), followed by Real time RT-PCR (Supplementary Fig. S7C, D). The results showed the infections could be efficiently detected at both 6 hours and highly increased at 48 h. However, we also found that Western Blotting methods were less efficient particularly for early infection detection of E and M proteins (Supplementary Fig. S8). We next analyzed cilia morphologies, and indeed observed a significant decrease in cilia size, including height, surface area and volume, in the E group and, to a lesser extent, the M group (Supplementary Fig. S9).

Cilia are assembled and disassembled during the G0 and G1 stages of the cell cycle [25, 26]. To further understand the cell cycle stage at which cilia are affected, we further adopted the Ki67 Fluorescent ubiquitination-based cell cycle indicator (FUCCI) cell cycle indicator system [27] (Fig. 3D). The efficiency of the infection was checked with counter staining of Strep Tag II antibodies (Supplementary Fig. S10). In combination with staining for the cilia marker acetylated alpha tubulin, we observed that the E protein could reduce the size of cilia at both the G0 and G1 stages (Fig. 3E, F and Supplementary Fig. S11A, B). Interestingly, we observed a significant decrease in the number of cells in the G2 stage but an increase in the number of cells in the G1 stage (Fig. 3G–I), suggesting that SARS-CoV-2 structural proteins could block the G1 to G2 transition.

Unexpectedly, in the infected cells, we also observed a very significant increase in the number of micronuclei in the E group at both time points (Fig. 3J–L), and an increase in the M group after 48 h. Concomitantly, the expression of p21, p27, p53 and RB1, the key cell cycle regulators, were initially upregulated but was subsequently downregulated by the E, M and N proteins (Fig. 3M and Supplementary Fig. S11C), suggesting the ability of the E, M and N proteins to disturb the cell cycle and induce cell cycle arrest at early stage of the infection. At the same time, we have also overserved E protein could elevate cytokines, such as IL-1β, IL-6, and IL-8, and also PTGS2 expression, but only after 48 h (Supplementary Fig. S11D, E). Similarly, in the cultured mouse tongue epithelium, the number of multinuclei/micronucleus cells increased, particularly with E protein, within the suprabasal layer cells (Supplementary Fig. S11F–H). The in vivo and ex vivo results indicated that these proteins could cause aberrant cell accumulation and mitotic deregulation at the G1 phase of the cell cycle. Therefore, our results indicate that the E protein might be the key SARS-CoV-2 viral component involved in the induction of epithelial cell fate changes.

## SARS-CoV-2 E protein can also disturb epithelial stratification

**Fig. 4:**
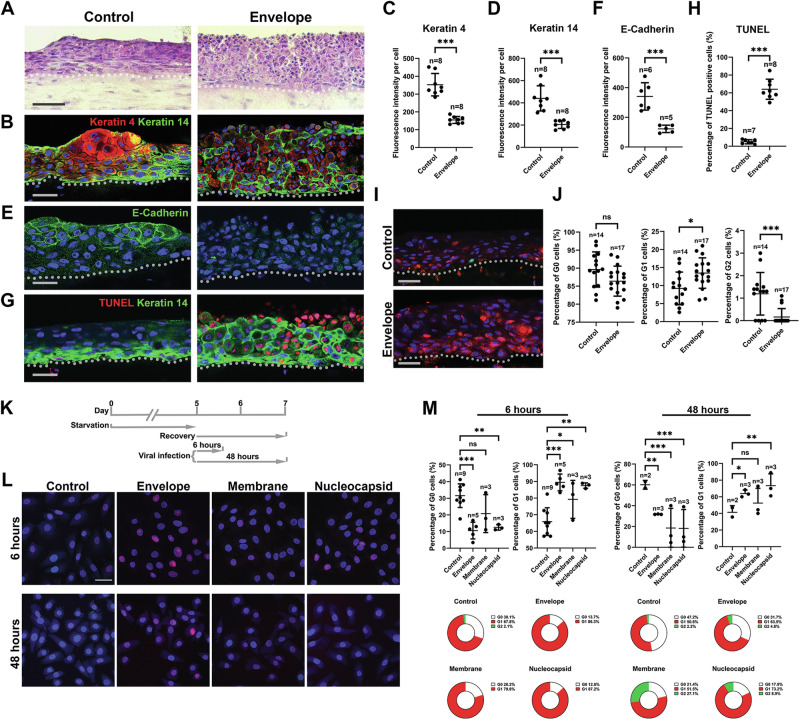
Envelope protein can disturb epithelial stratification by affecting cell cycle. **A**, **B**, **E**, **G** Representative histological and immunofluorescence analysis of the corresponding markers on the 3D human oral mucosa stratification model (also see Fig. 2D). **C**, **D**, **F**, **H** Quantification the signal of the indicated markers and positive cells (for TUNEL) in the corresponding conditions showed in (**B**, **E**, **G**). **I** Representative image for 3D human oral mucosa stratification model using HGEPp harboring Ki67-FUCCI system then infected with control or Envelope protein. **J** Quantification of the samples for conditions showed in (**I**). **K** Illustration of the cell cycle synchronization model. **L** Representative images for HGEPp (with Ki67-FUCCI) under synchronization and cell cycle re-entering model, at indication conditions. **M** Quantification of the cells showed in (**L**). Dotted lines indicate epithelial-mesenchymal junctions. *n* number of the image fields quantified. For statistical test results, center values represent mean and error bars represent s.d. ns no significance (*p* ≥ 0.05); *p < 0.05; **p < 0.01; ***p < 0.001. Bars: **A**: 100 μm; **B**, **E**, **G**, **I**, **L**: 20 μm.

In the stratified epithelium, cell differentiation is a highly organized event [28, 29]. Differentiation occurs during development and under wound healing conditions, normally through horizontal basal cell proliferation and vertical stratification, where cells move toward the basement membrane and undergo differentiation [30]. COVID-19 patients have impaired wound healing speed and ability [31]. COVID-19 manifestations include ulcerations in the oral cavity, throat and gastrointestinal ducts that are difficult to heal. In epithelial wound healing, the cells need to migrate into the wound and then undergo stratification to repair the damaged tissue [32]. To evaluate whether E protein can affect epithelial cell stratification, we further modified the three-dimensional human oral mucosa equivalents by infecting epithelial cells at the monolayer stage before stratification was initiated (Fig. 2D). Strikingly, when cells were exposed to the E protein at this stage (Supplementary Fig. S12), the epithelium structure was totally disorganized (Fig. 4A) and Keratin 4 (Fig. 4B, C) and E-Cadherin (Fig. 4E, F) expression was inhibited, and Keratin 14 expression was increased (Fig. 4B, D, G), indicating that cell differentiation was completely disrupted. The epithelial structures were filled with numerous apoptotic cells with abnormal nuclei cells surrounded by K14-positive cells (Fig. 4G, H).

We also established full-thickness human 3-dimensional equivalents and observed them using the Ki67-FUCCI system. The results again revealed increased numbers of G1-stage cells, notably in the suprabasal layer (Fig. 4I, J). To further confirm these observations, we performed a cell cycle synchronization experiment (Fig. 4K) in which the cells returned to the G0 stage and then re-entered the cell cycle (Fig. 4L, M). The results indeed revealed that when starved cells were released from the G0 phase, most of the E-affected cells entered but accumulated at the G1 phase. However, the disturbance of epithelial stratification can be affected by broader factors such as the increased epithelial apoptosis etc. that would need further investigation to prove the direction association of G1 arrest and stratification disturbance.

## CNN2 is a unique target of SARS-CoV-2 nonspike structural proteins

**Fig. 5:**
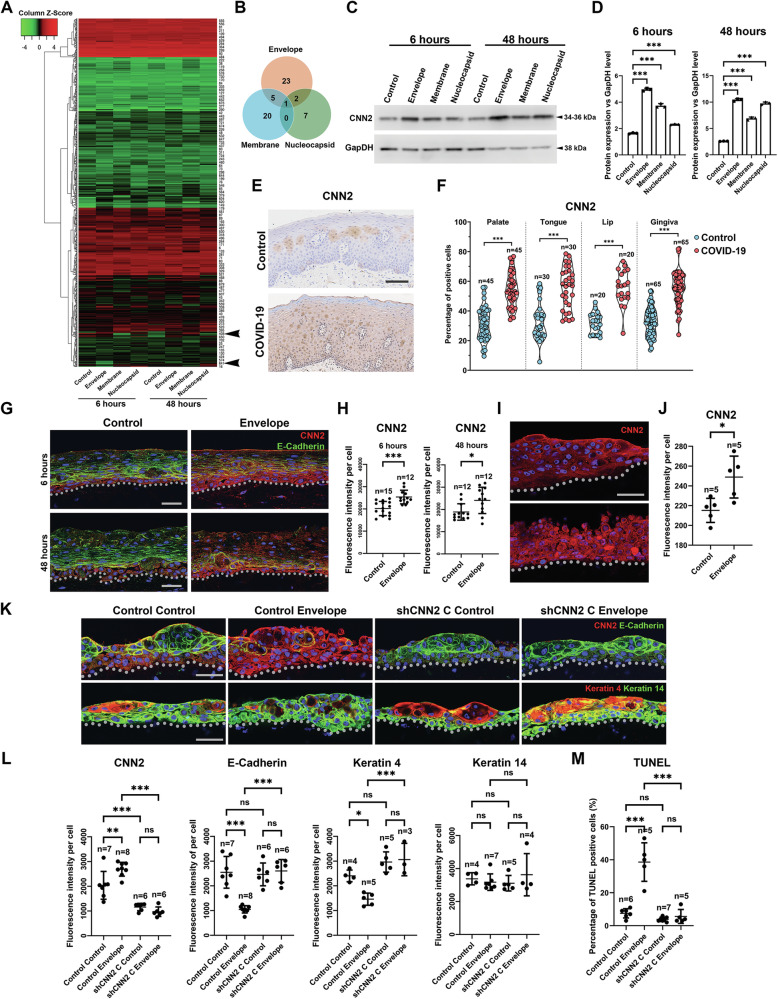
CNN2 is a functional target of SARS-CoV-2 nonspike structural proteins. **A** Bioinformatic analysis of the proteomic analysis results of HGEPp cells infected with indicated non-spike structural proteins. Arrows point to two clusters of molecules that were upregulated by the non-spike structural proteins. **B** Venn diagram of the molecules upregulated by the three proteins. Note there’s only one protein that was jointly upregulated, which is CNN2 (for details please see Supplementary Table 1). **C**, **D** Western Blotting and quantification of CNN2 in the indicated conditions and time points. **E** Representative images showing CNN2 expression in human oral mucosa in controls and COVID-19 patients. For additional images and lung and kidney analysis please see Supplementary Fig. S13. **F** Quantification of CNN2 positive cells in the oral mucosa. Immunofluorescence analysis of indicated markers in 3D human oral mucosa full-thickness infection (**G**, **H**) and stratification (**I**, **J**) models. **K**–**M** Representative immunofluorescent images for control or shCNN2 cells performed 3D stratification and infection model, for indicated markers, and TUNEL analysis. For shCNN2 selection and TUNEL images please see Supplementary Fig. S14. Dotted lines indicate epithelial-mesenchymal junctions. *n* number of the image fields quantified. For statistical test results, center values represent mean and error bars represent s.d. ns no significance (*p* ≥ 0.05); *p < 0.05; **p < 0.01; ***p < 0.001. Bars: **E**: 100 μm; **G**, **I**, **K**: 20 μm.

To explore the molecular mechanisms by which SARS-CoV-2 structural proteins affect human epithelial cell fate, we next performed proteomic analysis on nonspike structural protein-affected cells. The results showed that only a relatively small number of proteins could be differentially regulated by the E, M, and N proteins (Fig. 5A, and Supplementary Table 1). Interestingly, the E, M and N proteins shared a common target, Calponin 2 (CNN2), an actin filament-associated mechanoregulator [33] (Fig. 5B). Its differential expression was further validated by western blotting, where the E protein group samples presented the most significant upregulation (Fig. 5C, D). Elevated CNN2 levels could also be observed in the oral mucosa of COVID-19 patients (Fig. 5E, F and Supplementary Fig. S13A), and was verified in human oral mucosa 3D equivalents and stratification models (Fig. 5G–J), notably in the suprabasal layer cells. Importantly, the upregulation of CNN2 could also be identified in COVID-19 patient lung and kidney samples (Supplementary Fig. S13B–D). To understand the function of elevated CNN2 levels in the epithelium, we introduced shCNN2 into gingival epithelial cells to knock down CNN2 before introducing the E protein and cell stratification. The results showed that when CNN2 was knocked down (Supplementary Fig. S14A–D), it could counteract the effects of the SARS-CoV-2 E protein by maintaining Keratin 4, Keratin 14 and E-Cadherin expression (Fig. 5K, L and Supplementary Fig. S14D), and inhibiting apoptosis (Fig. 5M and Supplementary Fig. S14E).

## Glis2 is the key mediator of CNN2 expression

**Fig. 6:**
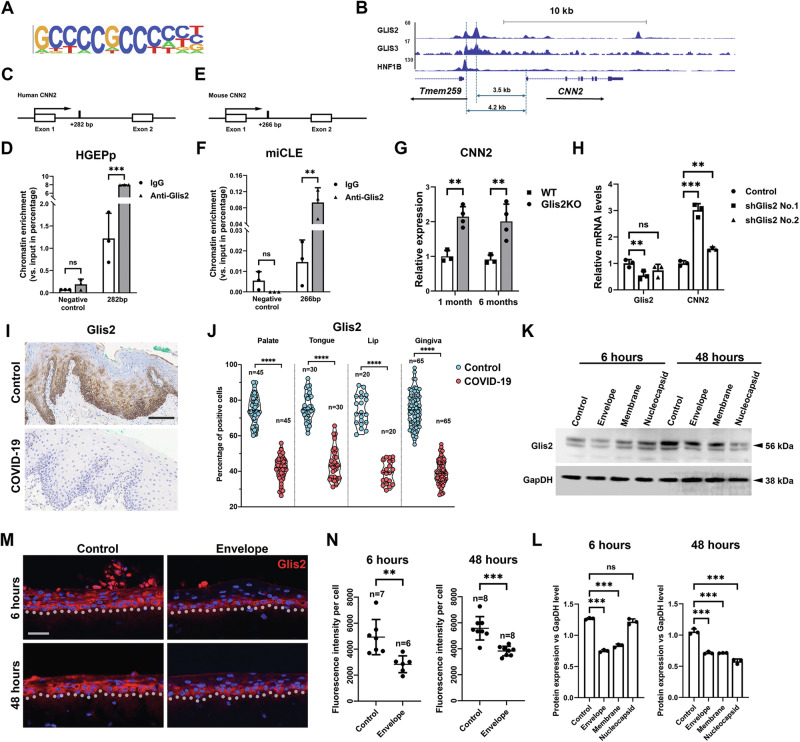
Glis2 is the key transcriptional factor mediating CNN2 expression. **A** Glis2 binding sequence used for ChIP-seq. **B** ChIP-seq results for potential binding peaks of Glis2 and Glis3 at CNN2 promoter and neighboring regions. **C**, **E** Illustration of predicted binding site of Glis2 at the first intron of human and mouse CNN2 genes. **D**, **F** Chromatin IP analysis of Glis2 binding to the sites on CNN2’s first introns showed in (**C**). **G** Real-time RT-PCR analysis of CNN2 expression in mouse kidney samples. **H** Glis2 and CNN2 expression analysis in control and shGlis2-treated miCLE cells. **I**, **J** Representative images showing Glis2 expression in human oral mucosa in controls and COVID-19 patients and quantifications. For additional images and lung and kidney analysis please see Fig. S15. **K**, **L** Western Blotting and quantification of Glis2 expression at the indicated conditions and time points. **M**, **N** Immunofluorescence analysis and quantification of Glis2 in 3D human oral mucosa full-thickness infection. Dotted lines indicate epithelial-mesenchymal junctions. *n* number of the image fields quantified. For statistical test results, center values represent mean and error bars represent s.d. ns: no significance (*p* ≥ 0.05); *p < 0.05; **p < 0.01; ***p < 0.001. Bars: **I**: 100 μm; **M**: 20 μm.

Sonic Hedgehog (Shh) is the most prominent pathway downstream of ciliary dynamics. To understand how CNN2 expression can be induced by SARS-CoV-2 nonspike structural proteins, we screened the CNN2 promoter, particularly the Shh pathway transcription factor-binding sites. We determined that GLIS2 was the sole core transcription factor for the pathway and had several potential binding sites on the CNN2 promoter. Chromatin immunoprecipitation (ChIP) sequencing analysis revealed several binding sites of GLIS2 in the promoter region and the first intron of the mouse CNN2 gene (Fig. 6A, B). Chromatin IP analysis using mouse tooth epithelial cells and human gingival epithelial cells further confirmed that GLIS2 indeed could bind specifically to the binding sequences located inside the first intron regions of the mouse and human CNN2 genes (Fig. 6C–F). GLIS2 can function as a repressor of gene expression [34]. Real time RT‒PCR analysis of wild type and Glis2 knockout (KO) kidney samples confirmed that, in Glis2 KO cells, CNN2 gene expression was highly elevated (Fig. 6G). Similarly, knocking down Glis2 with shRNA in mouse tooth epithelial cells also upregulated CNN2 expression (Fig. 6H). Therefore, GLIS2 appears to be a suppressor of CNN2.

We next investigated GLIS2 expression in the oral mucosa of COVID-19 patients and found that GLIS2 expression was indeed significantly downregulated (Fig. 6I, J and Supplementary Fig. S15A). Moreover, in SARS-CoV-2 nonspike structural protein-treated human gingival epithelial cells, Glis2 expression was suppressed by all three nonspike structural proteins (Fig. 6K, L). Similarly, in human oral mucosa 3D equivalent samples, Glis2 expression was downregulated by the E protein (Fig. 6M, N). Finally, we confirmed that Glis2 expression was downregulated in COVID-19 patient lung and kidney samples (Supplementary Fig. S15B–E).

## Discussion

Epithelial cell dedifferentiation has been observed mainly in the contexts of tissue regeneration and cancer [18, 19, 28, 29]. However, in the context of infectious diseases, it has been observed only in in vitro models [13, 20, 35]. Our study provides robust evidence that SARS-CoV-2 nonspike structural proteins that cause epithelial cell dedifferentiation and damage to different tissues share a similar novel molecular axis and a possibly “double hijack” mechanism: inducing dedifferentiation and at the same disturbing stratification (Supplementary Fig. S16). Nonspike structural proteins can cause aberrant epithelial dedifferentiation in differentiated cells and simultaneously impede epithelial progenitor cell stratification and terminal differentiation. Both cellular events result in cell cycle arrest at the G1 stage. Cell cycle arrest at the G1 stage normally prevents cells from replicating damaged DNA [36, 37]. However, we observed increased chromosome instability, apoptosis and cytokine production at the G1 stage, indicating that further damage occurred. Previous reports have shown that SARS-CoV-2 infection can induce DNA damage through CHK1 degradation and impair 53BP1 recruitment and cellular senescence [12]. It would be essential to understand if the same molecular mechanisms occurred exclusively in the G1 stage, through nonspike structural proteins.

Among the three SARS-CoV-2 nonspike structural proteins, the E protein showed the most potent ability to induce epithelial cell dedifferentiation, micronuclei formation, and ciliary abnormalities. Indeed, the E protein alone can cause acute respiratory distress syndrome (ARDS)-like pathological damage and constitute an antiviral target [38–40]. The E protein can form cation-conducting channels within the endoplasmic reticulum Golgi intermediate [41], where it serves as the center for cilia assembly and disassembly. The SARS-CoV-2 whole virus can damage the ciliary layer of tract cells by inhibiting key ciliogenesis regulators, such as Foxj1 and RFX3 [20]. We observed that, particularly in the E protein group, the epithelial cilia size was significantly reduced at both the G0 and G1 stages, especially when G0 to G1 transition was enforced, suggesting that the ciliary machinery might be disturbed at the disassembly stage. We therefore aimed to understand whether Foxj1 and RFX3 inhibition by SARS-CoV-2 is E protein dependent and whether it is cell cycle phase specific.

Our results revealed that all three nonspike structural proteins could decrease GLIS2 expression and increase CNN2 expression. We suggest that the E protein regulates the GLIS2-CNN2 signaling axis in cilia via Shh pathway modulation. However, as the M and N proteins do not cause significant changes in the cilia, other molecular regulatory machinery might also be involved. Interestingly, GLIS2 downregulation has been associated with lung and liver, and kidney fibrosis [42–44], and CNN2 downregulation has been linked with kidney fibrosis [45], both of which are also key pathological features of COVID-19. Essentially, we discovered that knocking down CNN2 could counteract the effects of the E protein on epithelial cell differentiation, stratification, and apoptosis, suggesting that CNN2 might be a novel therapeutic molecular target for COVID-19.

## Materials & methods

### Human tissues

For dedifferentiation, Glis2 and CNN2 analysis, the human tissues were achieved from Brazil and approved by the Brazilian National Research Ethics Committee (CONEP #60945122.4.1001.5418). The samples were collected from biopsies and autopsies at three oral diagnosis centers (Getulio Sales Diagnosticos, Brazil; AC Camargo Center, São Paulo, Brazil; Universidad Nacional Autónoma de México/UNAM, Mexico City, Mexico, details please see Supplementary Table 2). Biopsies from Mexico were collected and sent to Brazil through a Material Transfer Agreement (MTA). All patients signed the Informed Consent Form (ICF). Biopsies were collected between 2020 and 2022 from patients presenting with chronic ulcers and non-healing lesions. Sample distribution included gingiva (15 COVID + 15 controls), tongue (12 COVID + 12 controls), palate (8 COVID + 8 controls), and buccal mucosa (7 COVID + 7 controls). Control samples were obtained from patients with inflammatory fibrous hyperplasia or other reactive/inflammatory oral mucosal conditions prior to 2016, matched by age and sex. Inclusion criteria for COVID-19 patients: confirmed RT-PCR diagnosis, presence of oral lesions resistant to conventional treatment including topical corticosteroids, and no history of other systemic diseases affecting oral mucosa. Control patients were selected based on absence of COVID-19, similar reactive/inflammatory lesions, and matched demographic characteristics. A total of 64 patients’ oral cavity samples were included in this study: 32 COVID-19 patients and 32 matched controls. COVID-19 diagnosis was confirmed by oronasopharyngeal swab RT-PCR (Xpert Xpress SARS-CoV-2). Viral RNA presence was additionally confirmed in tissue samples. Kidney and lung samples were collected from autopsies of patients with severe COVID-19 (*n* = 5 for each tissue) who developed acute respiratory distress syndrome and multiple organ failure, leading to death. All patients had COVID-19 diagnosis confirmed by RT-PCR from nasopharyngeal swabs during hospitalization, and viral RNA was additionally confirmed in the FFPE tissue samples using RT-PCR. Control samples (*n* = 5 for each tissue) were obtained from non-neoplastic tissue adjacent to tumors from kidney and lung biopsies, ensuring maximum distance from tumor margins. All tissues were properly fixed in buffered formalin and processed using automated histological processing to ensure sample conformity.

For ACE2 and TMPRSS2 analysis, the samples were collected at Nanjing Medical University Affiliated Stomatological Hospital with approval received from the Ethics Committee of Nanjing Medical University Affiliated Stomatological Hospital (PJ2022-191-001). Human tongue normal tissue biopsies were collected between 2019 and 2020 from patients bearing tongue squamous cell carcinoma (*n* = 8), matched by age and sex.

### Mouse strains

All the animal breeding and procedures were approved by the institutional animal care and use committees at individual universities and in accordance with the guidelines and regulations for the care and use of laboratory animals in the corresponding countries: CD1, C57BL/6 mice at the University of Plymouth, UK. The Glis2-HA mice (C57BL/6J-Glis2<tm3Amj>) from NIH was generated by inserting HA tag into exon 7 of Glis2 right before the TGA stop codon, thereby generating a GLIS2-HA fusion protein. Animal studies followed the guidelines outlined by the NIH Guide for the Care and Use of Laboratory Animals and protocols were approved by the Institutional Animal Care and Use Committee at the National Institute of Environmental Health Sciences. No statistical methods were used for estimating sample size.

### Mouse tongue organ culture

Mouse tongues were dissected from postnatal 2-month-old C57BL/6 mice. Tongues were cultured in 24 well plate (Greiner, 662641) with 1 ml DMEM/F12 containing 20% FBS with 1% penicillin-streptomycin and 1% Amphotericin B (Gibco, 15290-026) overnight before conducting viral infection. Samples were infected with 500 μl lentiviral particles. After 2 h, fresh normal culture medium was topped up. Samples were frozen directly in Tissue-Tek O.C.T. compound (Sakura Finetek) and sectioned at 20 μm for further analysation.

### Cell culture

Human gingival epithelial cells (HGEPp, CELLnTEC) were cultured in CnT-57 (CELLnTEC). Human gingival fibroblasts (HGFs) were purchased from Innprot (Cat. P10866) and cultured in DMEM/F12 (Gibco, 31331-028) containing 20% Fetal bovine serum (FBS) (Sigma, F7524), and 1% penicillin-streptomycin (Hyclone, SV30079.01). Human embryonic kidney 293 cells (HEK293FT) were cultured in DMEM (Gibco, 31966-021) containing 10% FBS and 1% penicillin-streptomycin. Mouse incisor cervical loop epithelial cells (miCLE) [27] were cultured in DMEM/F12, which combines B-27® supplement (Gibco, 12587-010), mouse epidermal growth factor (EGF) (Bio-techne, 2028-EG-200), mouse fibroblast growth factor 2 (FGF2) (Bio-techne, 3139-FB-025/CF) with 1% penicillin–streptomycin. For all cell lines, passage 3–7 cells were used for further experiment and investigation.

### Collagen gel-based full-thickness human oral mucosa 3D equivalents

For each 3D equivalent gel, 5 × 10^4^ of HGFs were mixed with 100 μl gel containing rat tail collagen (Fisher, 11519816), 10× DMEM (Fisher, 21969-035) and FBS at a ratio of volume 9:1:1. The mixture was mixed on ice with 1.6 μl 1 M Sodium hydroxide solution (NaOH) (Sigma, 71687) and 4 μl 7.5% Sodium bicarbonate (NaHCO_3_, Sigma S6297) for crosslinking. The gel was then pipetted into a 0.4 μm culture insert (Merck, PIHP01250) and incubated at 37 °C for 1 h followed by adding fresh culture medium was added into the insert. Culture medium was replaced by CnT-57 before seeding 1–1.5 × 10^6^ of HGEPp on top of the gel. HGEPp was cultured for 72 h before airlifting then culture medium was change to CnT-3D (Caltag, CnT-3D). Culture medium inside the insert was removed every day and the medium outside was changed every 2 days. Samples were frozen directly in OCT (Agar Scientific, AGR1180) at different time points and sectioned at thickness of 20 μm for further analysis. 3D equivalent with Ki67-FUCCI HGEPp was fixed with 4% PFA (Sigma Aldrich, 158127) for 30 min and went through serial dilution of sucrose (10%, 20% and 30% sucrose for 15 min each), before freezing.

### Generation of lentiviruses, retroviruses and cell infection

Lentiviral plasmids for SARS-CoV-2 non-spike structural proteins were purchased from Addgene [23] (for details, please see Supplementary Table 3). Lentiviruses were prepared using JetOPTIMUS (Polyplus) mediated transfection on HEK293FT cells. Lentiviral supernatant was collected according to the manual using HEK293FT cells. HGEPp were infected with 1 ml lentiviruses carrying the target sequences above with 10 μg/ml polybrene (Merck, TR-1003). After 2 h, dishes were topped up with 3 ml fresh culture medium. Samples were collected at 6 h or 48 h.

Ki67p-T2A-FUCCI virus were prepared as previously described [27]. HGEPp was incubated with 1 ml lentiviruses containing supernatant with 10 μg/ml polybrene for 2 h. Following the incubation, the viral supernatant was replaced with 3 ml normal growth media. Infected cells were selected using medium with 10 μg/ml blasticidin (Sigma Aldrich, 15205).

Transfection of pLKO.1 (control), pLKO.1-shGlis2 (No.1 and 2) and cell infection were performed as previously described [27]. shCNN2 were purchased from Origene (details please see Supplementary Table 3). Retroviruses were prepared using JetOPTIMUS (Polyplus) mediated transfection of PLAT-A cells. Retroviral supernatant was collected according to the manual using PLAT-A cells. HGEPp were infected with 1 ml retroviruses carrying the target sequences above with 1 μl 10 μg/ml polybrene (Merck, TR-1003). After 6 h, dishes were changed into 3 ml fresh culture medium. Infected cells were selected under 1 μg/ml puromycin for 7 days. Cells were collected for validation and further experiments.

For full thickness 3D equivalents, samples were incubated with 200 μl lentiviral particles with 10 μg/ml polybrene (Merck) at day 14 day after airlifting. After 2 h exposure to the virus, inserts were topped up with 300 μl inside and 3 ml outside of fresh normal culture medium. Samples were collected at 6 h and 48 h after infection.

For 3D stratification models, samples were incubated with 200 μl lentiviral particles carrying target sequences above with 10 μg/ml polybrene (Merck) after HGEPp cell seeding. After 2 h, inserts were topped up with 300 μl inside and 3 ml outside of fresh normal culture medium. Virus was removed and changed to fresh culture medium after 24 h. Samples were cultured till defined time points for analysis.

### Cell cycle synchronisation experiments

24 h after initial seeding and culturing under normal cell culture conditions, HGEPp carrying Ki67p-T2A-FUCCI were exposed to basal medium (DMEM). After 5 days, cells were infected with 500 μl lentiviruses carrying the target sequences above with 10 μg/ml polybrene (Merck, TR-1003) for 2 h, followed by topping up with 1 ml fresh CnT-57 culture medium.

### Haematoxylin & Eosin staining

Frozen slides were fixed in 4% PFA for 30 min then briefly washed in distilled water before being stained for 3–8 min in Harris Haematoxylin (Sigma, HHS16). After staining was sufficient the slides were washed in tap water for 5 min. Differentiation of the stain was achieved by placing the slides in 1% Acid alcohol (Sigma Aldrich, 56694) for 30 s, before a further 5-min tap water wash. Subsequent counter staining was performed by washing the slides briefly in 95% alcohol, followed by a 1.5 min incubation in 0.25% eosin Y solution (Sigma, 230251). After staining was complete, the tissue was dehydrated by passing the slides through two 5-min washes in 95% alcohol, then 100% alcohol. the slides were incubated in xylenes for 5 min before being mounted with Eukit (Fluka, 03989).

### Immunohistochemistry and immunofluorescent analysis

Immunohistochemistry and immunofluorescent analysis were performed single blindly between Professor Bing Hu, Professor Ciro Dantas Soares and Professor Jinhua Yu’s groups without previously knowing each other’s experimental results.

#### Group Bing Hu (Plymouth)

Preparation and fixation of cells to be used for immunofluorescent (IF) analysis was performed. Cells were washed in HBSS (Gibco, 14175-053). Then fixed in ice cooled 4% PFA (Sigma Aldrich, 158127) solution in 10 mM PBS (Sigma, P4417) for 30 min. Frozen tissue was cryosectioned at 20 μm thickness on a Leica CM1850 cryostat. Sections were mounted onto Polysine™ Microscope Adhesion Slides (Thermo Scientific, J2800AMNZ) and allowed to air dry for 30 min before were fixed in freshly made ice cooled 4% PFA solution in 10 mM PBS for 30 min. Once prepared all sample types were washed three times in PBST (PBS containing 0.1% Triton-X100, (Sigma, X100)) for 5 min per wash. Non-Specific binding was blocked by incubation for 60 min with PBST containing 5% Donkey Serum (Sigma, D9663). Primary antibodies were incubated overnight (for details please see Supplementary Table 3). Slides were washed three times in PBST at room temperature before incubation with secondary antibodies for 2 h at room temperature. Secondary antibodies were incubated for 2 h. Nuclei were counterstained with 2 μg/ml DAPI (Sigma, D9542) for 10 min. Samples were mounted with DAKO (Align, DAKO) mounting medium and sealed with nail polish. IF images were captured using a Leica DMI6000 confocal microscope with a Leica TCS SP8 attachment. The microscope is running LAS AF software from Leica. Images for comparison were taken using the same settings and post imaging processing was conducted using Adobe Photoshop CC also in parallel between comparable samples.

#### Group Soares (Brazil)

Four-micrometer-thick sections obtained from FFPE blocks were stained using the Autostainer Link 48, Dako platform, with FLEX detection. For the antibodies details please see Supplementary Table 3. Slides were digitally scanned using an Aperio® AT2 microscope slide scanner (Leica) at 40× magnification. Images were independently analyzed at Soares’ group. Quantitative analysis was performed using ImageScope software (Version 12.4.3.5008, ImageNav 12.4.3, Viewport 12.4.0/12.4.5). Ten representative high-power fields (40×) were analyzed per sample. Total cell count and positive cell count were performed for each field, generating a percentage value per marker per patient.

#### Group Jinhua Yu (Nanjing)

Preparation of formalin-fixed paraffin-embedded samples were sectioned at a thickness of 4 μm, and mounted onto Superfrost slides (Sigma Aldrich, Z692255). After drying overnight. Slides were deparaffinized by heating to 60 °C for 1 hour before three times washing in xylenes (Sigma Aldrich, 534056) for 10 min. Slides were then washed in 100% industrial methylated spirits (IMS) (VWR, 23684.360) for 5 min, before being washed for 5 min in 95%, 85% IMS and then 75% IMS. Antigen retrieval was performed by boiling the slides in a 0.01 M citrate buffer solution (citric acid and 0.05% Tween-20, Sigma Aldrich, C2404 and P9416, pH6.0) for 15–20 min. Non-specific binding was blocked by incubation with blocking buffer containing 10% donkey serum for 30 min. Primary antibodies diluted in blocking buffer were incubated overnight at 4 °C. Slides were washed thrice in PBS before incubation with secondary antibodies for 20 min at room temperature. DAB was performed for several minutes then stopped by wash by running tap water. Nuclei were counterstained with haematoxylin for 20 s to 2 min. Slides were mounted by neutral balsam. Images were captured using a Olympus BX51 with Olympus VS200 attachment.

### Three-dimensional (3D) reconstruction and Cilia quantification

Z-stack images of immunofluorescence staining acquired from Leica confocal microscope were used to reconstruct primary cilia in 3D using Imaris 9.6.0 (Bitplane, 9.6.0). Measurements of primary cilia dimensions were performed manually using the Surface and Measurement tools in Imaris for further quantifications.

### Terminal deoxynucleotidyl transferase dUTP nick end labeling (TUNEL)

Samples were washed briefly 5 s in PBS twice, then fixed in 4% PFA for 30 min then washed 2 times in PBS 5 min each. Samples were permeabilized by PBST for 2 min on ice then incubated in 20 μl TUNEL mixture pre section (In Situ Cell Death Detection Kit, TMR red, 12156792910, Roche) for 4 h at 37 °C in humid atmosphere. After the washing and mounting, images were captured using a Leica DMI6000 confocal microscope with a Leica TCS SP8 attachment.

### Real-time RT-PCR and data analysis

Real-time RT-PCR analysis was performed on a LightCycler 480 Real Time system (Roche) for 45 cycles, using a SYBR Green I MasterMix (Roche, 04887352001) and primers (for details, please see Supplementary Table 3). 36β4 gene and GapDH gene were used as housekeeping gene. Analyses were performed using three technical replicates using the 2^−ΔΔCt^ method as previously described [27].

For verifying the PCR product size, the products were run on an 2% agarose gel (2% Agarose (Thermo Fisher Scientific, BP160-100) in TAE buffer (Life Technologies, 15558-042)). Once microwaved and cooled SYBR Safe DNA gel stain (Invitrogen, PIN533102) was added to the molten gel before being cast. Following pouring the gel was allowed to set for 30 min at room temperature. The gel was submerged in TAE buffer and the comb removed. 9 μl of PCR product was mixed with 1 μl of loading dye (Thermo Fisher Scientific, R0611) and loaded into each well. The gel was electroporated for 30 min at 110 volts at room temperature. The gel was then imaged using a D-Digit UV light chamber (LI-COR) with D-Digit Image Acquisition software (LI-COR).

### Western blotting

A NuPage® Electrophoresis System (Thermo Fisher Scientific), 4–12% Bis-Tris gradient gel (Thermo Fisher Scientific, NP0335BOX), and MOPS buffer (Thermo Fisher Scientific, NP0001). 25–40 μg protein were incubated at 95 °C for 5 min before loading for separation on a 4–12% Bis-Tris gradient gel (Thermo Fisher Scientific, NP0335BOX) with MOPS buffer (Thermo Fisher Scientific, NP0001) of NuPage® Electrophoresis System (Thermo Fisher Scientific). Transfer of protein samples onto a 0.45 μm PVDF membrane (Thermo Fisher Scientific, LC2005) was carried out using a NuPage® XCell II Blot Module, and transfer buffer (Thermo Fisher Scientific, NP0006) with 10% methanol (Sigma, 322415). The iBind™ Western System (Thermo Fisher Scientific) was used for blocking, primary and secondary antibody incubations (for details, please see Supplementary Table 3). A C-Digit scanner (LI-COR) was used for band detection with Image studio software (LI-COR, Version 3.1).

For blotting virus structural protein, 25–40 μg protein were loaded, but without 95 °C denaturing step. Protein separation was performed on a 4–12% Bis-Tris gradient gel (Thermo Fisher Scientific, NP0335BOX) with MES buffer (Thermo Fisher Scientific, NP0002) of NuPage® Electrophoresis System (Thermo Fisher Scientific). Transfer of protein samples onto a 0.22 μm PVDF membrane (Thermo Fisher Scientific, LC2002) was carried out using a NuPage® XCell II Blot Module, and transfer buffer (Thermo Fisher Scientific, NP0006) with 10% methanol (Sigma, 322415). The iBind™ Western System (Thermo Fisher Scientific) was used for blocking, primary and secondary antibody incubations (for details, please see Supplementary Table 3). A C-Digit scanner (LI-COR) was used for band detection with Image Studio software (LI-COR, Version 3.1).

## Proteomic analysis

### Sample preparation for mass spectrometry

HGEPp cells were seeded in 6 cm dishes and performed viral infection and protein treatment for 6 h and 48 h. To collect protein after each time points, culture media was removed from the culture dishes and cells were washed in pre-cooled HBSS twice. HBSS was removed and replaced with ice-cold RIPA buffer (ThermoFisher, 89901) supplemented with Halt™ Protease and Phosphatase Inhibitor Cocktail (ThermoFisher, 78440) at 1:100. The cells were detached from the dish using a cell scraper and then collected into an Eppendorf tube on ice then incubated for 30 min with frequent agitation for efficient cell lysis and solubilisation of proteins. Tubes were spun down at 15,000 rpm for 15 min at 4 °C so the supernatant containing the protein could be collected and stored at −80 °C until ready to load onto a gel. 15 µg protein samples were run on a Nupage 4–12% Bis-Tris protein gel (Invitrogen) at 200 V for 45 min. Gel was rinsed with water before fixing in 40% (v/v) ethanol and 10% (v/v) acetic acid for 15 min with gentle agitation. After washing the gel twice in water, the gel was stained overnight in QC colloidal Coomassie Blue G-250 (Biorad). The gel was destained for 1 h with changes of water every 15 min until protein bands were visible. Every sample lane was cut into 4 fractions and each fraction further cut into 1–2 mm cubes for proteomics analysis.

### Proteomic analysis

Sample preparation, in-gel digestion, sample cleanup and mass spectrometric analysis were carried out as previously described [11]. Briefly, the gel slices were incubated with trypsin overnight followed by peptide clean up using inhouse STAGE tips procedure. Peptides were then separated on a Vanquish Neo system (Thermo Fisher Scientific, UK). A 5 µl of sample was loaded in 0.1% FA (Formic Acid) and acetonitrile (2% acetonitrile in 0.1% FA) onto Easy Spray C18 nano column 75 µm × 15 cm, 3 µm (Analytical Column) with a linear gradient of 96% buffer A and 4% buffer B to 75% buffer A and 25% buffer B, at a constant flow rate of 300 nl/min over 90 min followed by ramping to 35% buffer B for 30 min. The sample was ionized in positive ion mode Easy spray ESI source (Thermo Fisher Scientific, UK) and analyzed in an Orbitrap Fusion Lumos mass spectrometer (Thermo Fisher Scientific, Bremen, Germany). The Orbitrap Fusion Lumos instrument under Xcalibur 4.4 software was operated in the data-dependent mode to automatically switch between MS and MS/MS acquisition. MS spectra of intact peptides (m/z 375–1500) with standard automated gain control accumulation with a resolution of 120,000. The most intense ions were sequentially isolated and fragmented in the quadrupole by Higher Energy C-trap dissociation (HCD) at a target value of 5000 or maximum ion time of 35 ms. A dynamic exclusion of ions previously sequenced within 50” was applied. All the singly charged and unassigned charge state ions were excluded from sequencing. Typical mass spectrometric conditions were: spray voltage, 1.8 kV; no sheath and auxiliary gas flow; heated capillary temperature, 275˚C; normalized HCD collision energy 30% for MS2 in Ion Trap. The ion selection threshold was 5000 counts for MS2.

### Data analysis

Peptides and proteins were identified by MaxQuant (2.4.11.0) using automated database searching of all tandem mass spectra against a curated target/decoy database (forward and reversed version of the *Homo Sapiens*, downloaded May 2022). Spectra were initially searched with a mass tolerance of 6 ppm in MS mode and 0.5 Da in MS/MS mode and strict trypsin specificity and allowing up to 2 missed cleavage sites. Cysteine carbamidomethylation was searched as a fixed modification, whereas N-acetyl protein, deamidated NQ, oxidized methionine were searched as variable modification. The resulting peak list-output files were automatically loaded into inbuilt software modules for further processing, label-free quantitation (LFQ) and a maximum false discovery rate of 1% was fixed for the result output files. The mass spectrometry proteomics data have been deposited to the ProteomeXchange Consortium via the PRIDE partner repository with the dataset identifier PXD060880. Proteomics data visualization and clustering were conducted with Genepattern [46].

### GLIS2 ChIP-Seq

ChIP-Seq was performed using primary renal epithelial cells (RECs) from *Glis2-*HA mice. Primary RECs were isolated as described previously [47]. The isolated cells were crosslinked in 1% formaldehyde for 10 min then and quenched by the addition of 125 mM glycine for 10 min. ChIP was performed as previous report [48]. HA antibody (#3724, Cell Signaling) was used to pull down GLIS2-HA-bound chromatin. DNA sequencing library was generated with the ChIPed DNA using a NEXTflex Rapid DNA sequencing kit (Bioo Scientific). Sequencing reads were obtained using a MiSeq Sequencing System (Illumina).

### Chromatin immunoprecipitation

Potential Glis2 transcription factor-binding sites in the CNN2 promoter were determined using CiiiDER software. Primers were designed using Primer3 and validated by RT-PCR. ChIP assays were performed using ChIP-IT High Sensitivity kit (Active Motif, 53040) following the kit’s manufacture. In brief, after crosslinking, nuclei were purified and chromatin was sheared by sonication using Bioruptor Pico sonicator (Diagenode) at frequency of 20 times, 15 s ON/30 s OFF to achieve enriched fragments. Sheared chromatin was incubated overnight with antibody directed against Glis2 (AVIVA, ARP30037_P050). Matching rabbit IgG Isotype antibodies were used as a negative control for the immunoprecipitation. Immunoprecipitated chromatin was then incubated with agarose beads, washed, and eluted. After reversal of the crosslinks and purification of DNA, precipitated DNA was analyzed by RT–qPCR. ChIP was performed on cells using the ChIP-IT high sensitivity kit (Active Motif, 53040) in accordance with the provided protocol. In summary, the procedure was as follows; cells were fixed using Complete Cell Fixation Solution added directly to the culture media for 15 min at room temperature. Fixation was quenched using Stop Solution. Cells were scraped and collected by centrifugation at 1250 r.c.f. for 3 min at 4 °C. The pellet was resuspended and washed twice in ice-cold PBS. The resultant pellet was resuspended in Chromatin Prep Buffer supplemented with protease inhibitor cocktail (PIC) and phenylmethylsulfonyl fluoride (PMSF) and incubated for 10 min on ice. Using a Dounce homogenizer the cells were homogenized, collected by centrifugation, and resuspended in fresh ChIP Buffer supplemented with PIC and PMSF. Chromatin was sheared using a Diagenode Bioruptor Pico (Diagenode) by sonicating for 15 s followed by 30 s of rest for 40 cycles. Cell debris was removed by pelleting this out by centrifugation. Antibodies or control IgG were mixed with Blocker as outlined in Supplementary Table 3; this antibody mix was mixed with ChIP Buffer, PIC, and sonicated chromatin and incubated overnight at 4 °C. Immunoprecipitation was performed by washing Protein G agarose beads in TE pH 8.8 and then mixing the washed beads to the antibody/chromatin mixture and incubating for 3 h at 4 °C. Following incubation with the beads the solution was passed through a ChIP filtration column to collect the bound chromatin. The bound chromatin was washed and eluted using the provided solutions. The cross-links within the eluted sample were reversed and the DNA purified by overnight incubation with Proteinase K at 37 °C. The DNA was collected by heating the sample to 95 °C for 8 min and then plunging into ice for 1 min to deactivate the proteinase K function. DNA Purification Binding Buffer and sodium acetate were added to the sample, which was then passed through a DNA Purification Column by centrifugation. The latter was washed with DNA Purification Wash Buffer, and DNA was eluted using DNA Purification Elution Buffer. Input DNA was prepared in the same manner resuspended in 0.1% diethylpyrocarbonate (Sigma, D5758)-treated distilled water (DEPC).

### Statistics

Statistical analyses were performed using Prism software (GraphPad software, Version 10.4.1). Unpaired *t*-test or One-way ANOVA were applied to all measurements. All quantification and Real time RT-PCR results were presented using style of mean and standard deviation (error bars). Observed differences were calculated for *p*-values: *p < 0.05; **p < 0.01; ***p < 0.001.

## Supplementary information

Supplementary Figure 1

Supplementary Figure 2

Supplementary Figure 3

Supplementary Figure 4

Supplementary Figure 5

Supplementary Figure 6

Supplementary Figure 7

Supplementary Figure 8

Supplementary Figure 9

Supplementary Figure 10

Supplementary Figure 11

Supplementary Figure 12

Supplementary Figure 13

Supplementary Figure 14

Supplementary Figure 15

Supplementary Figure 16

Original data file for Western Blot

Supplementary Table 1

Supplementary Table 2

Supplementary Table 3

Supplementary Figures Caption

## Data Availability

The datasets generated during and/or analyzed during the current study are available from the corresponding author on reasonable request.

## Data deposition

The ChIP-seq data can be accessed through number: GSE128234 from https://www.ncbi.nlm.nih.gov/geo/. The proteomic analysis data can be accessed from PRIDE (https://www.ebi.ac.uk/pride/) through number: PXD060880.
