# Supplementary figures and images for: SARS-CoV-2 nonspike structural proteins hijack mucosa epithelial cell fate
Source: Cell Death Dis. 2026 Mar 23;17(1):340. doi: 10.1038/s41419-026-08611-6 (PMC13039937; doi:10.1038/s41419-026-08611-6)

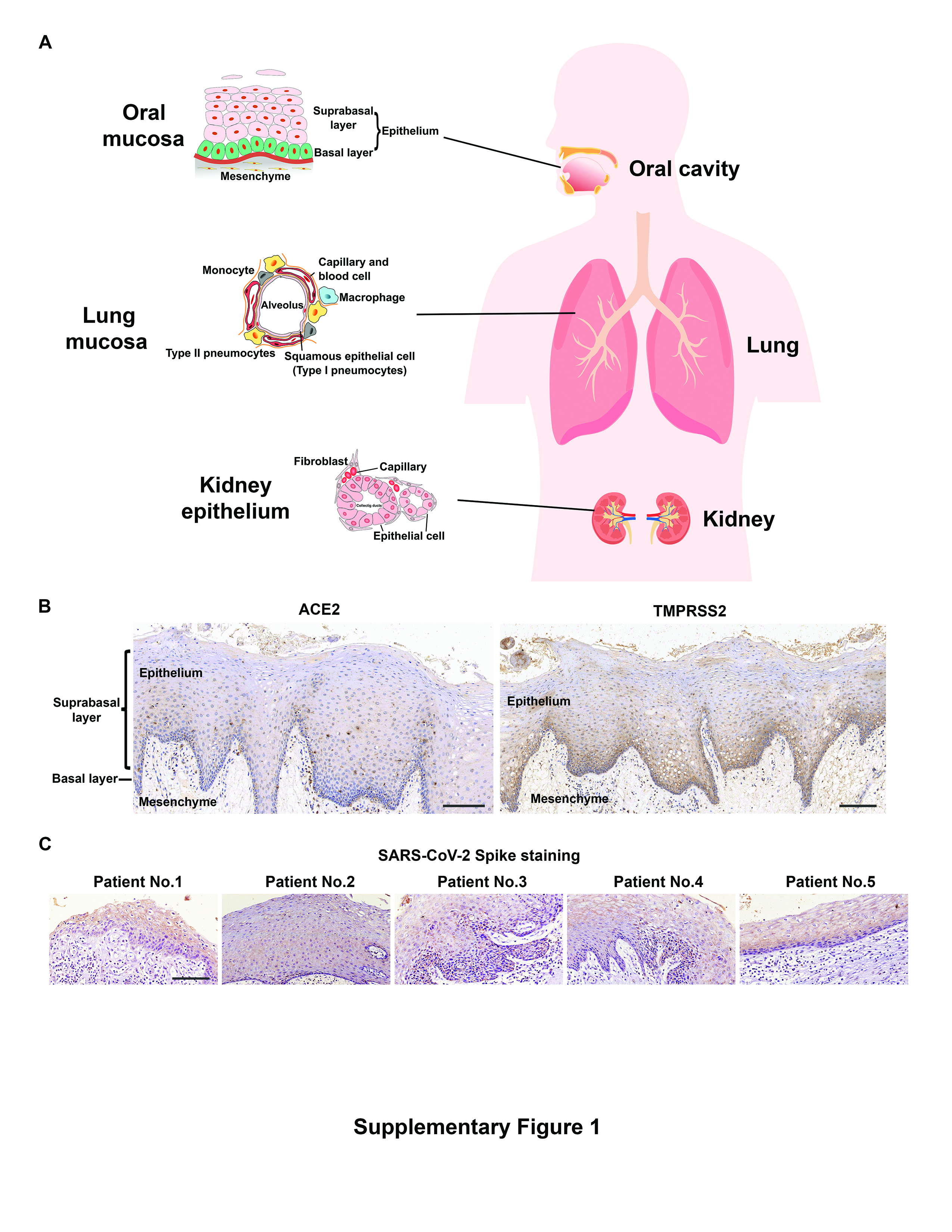

Supplement: Supplementary file 1 — Supplementary Figure 1 [file 41419_2026_8611_MOESM1_ESM.tif]

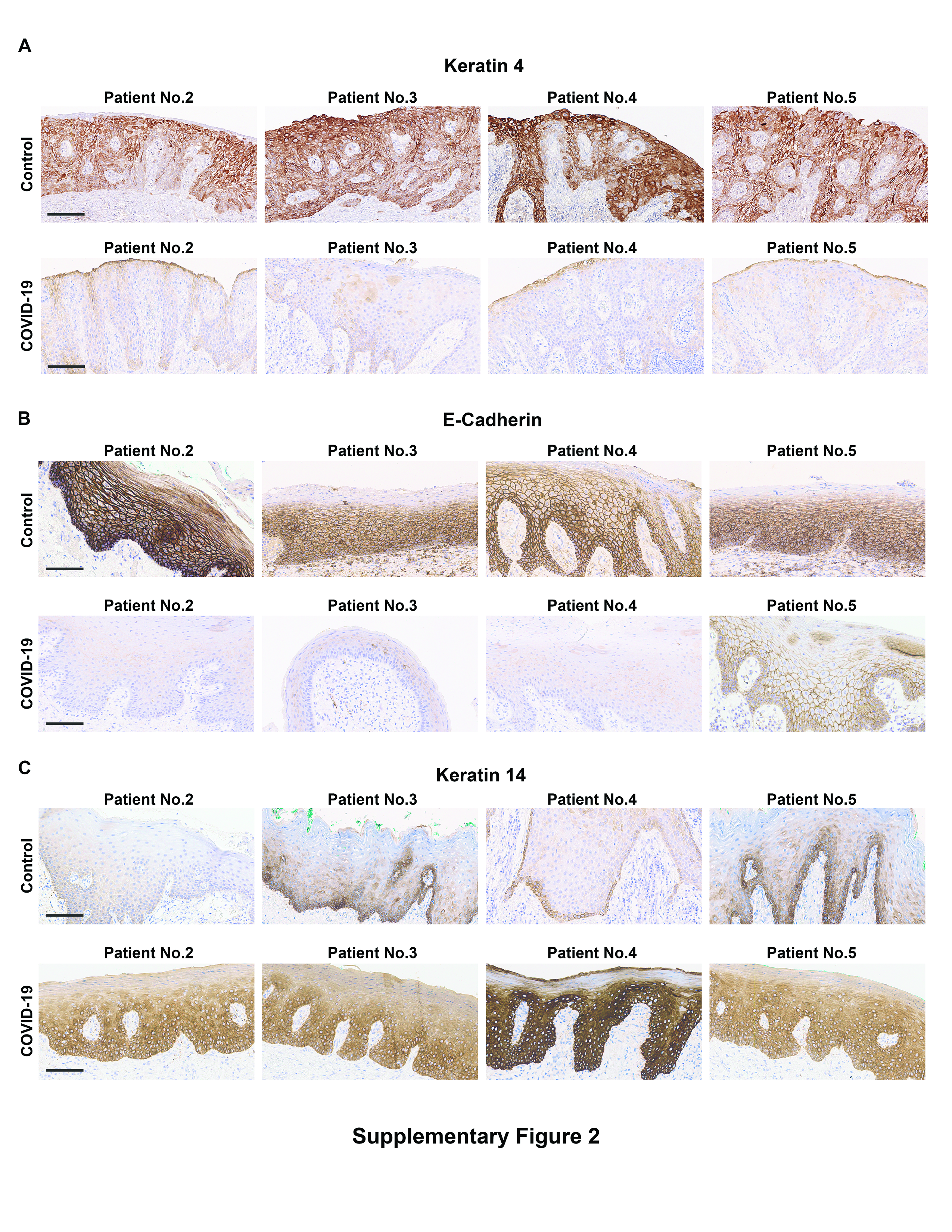

Supplement: Supplementary file 2 — Supplementary Figure 2 [file 41419_2026_8611_MOESM2_ESM.tif]

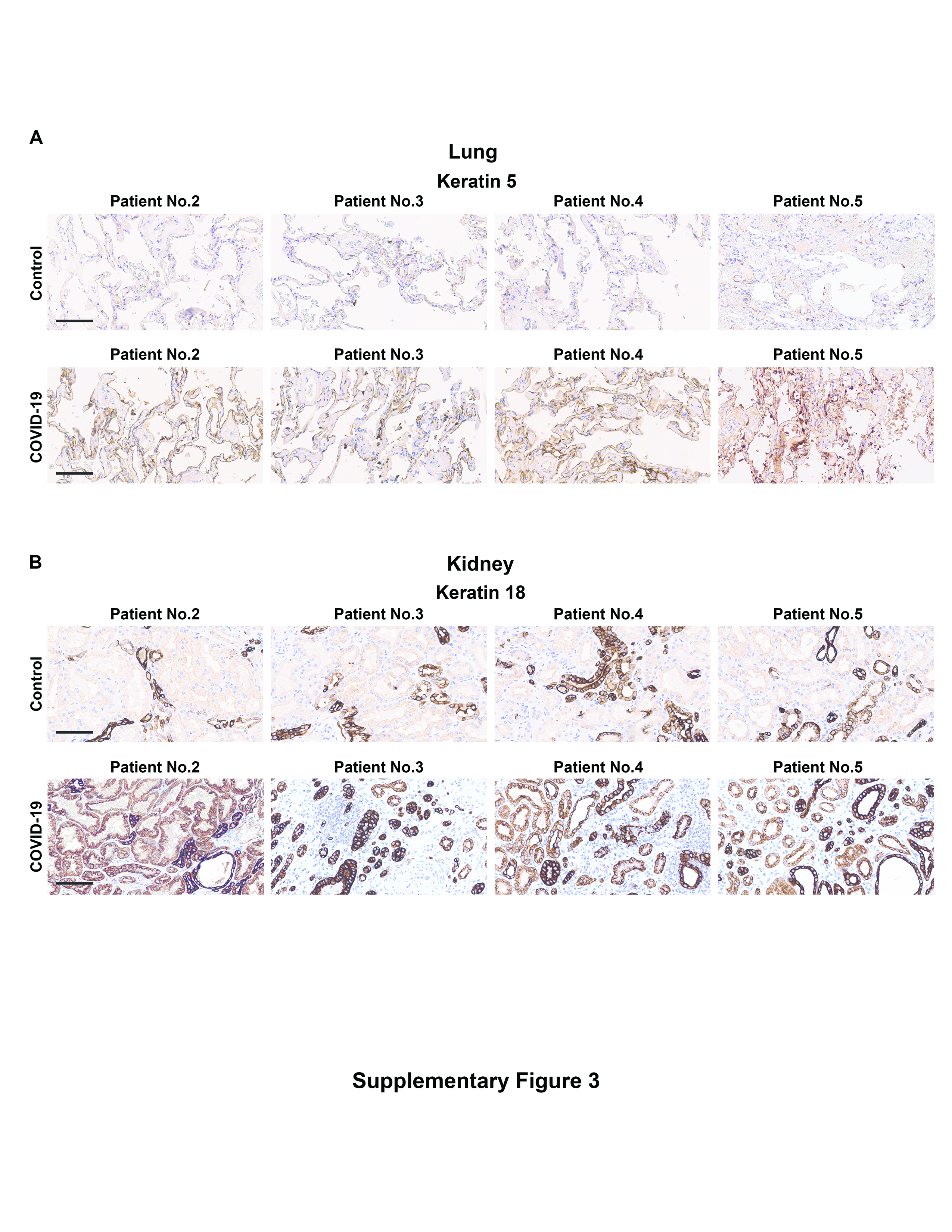

Supplement: Supplementary file 3 — Supplementary Figure 3 [file 41419_2026_8611_MOESM3_ESM.tif]

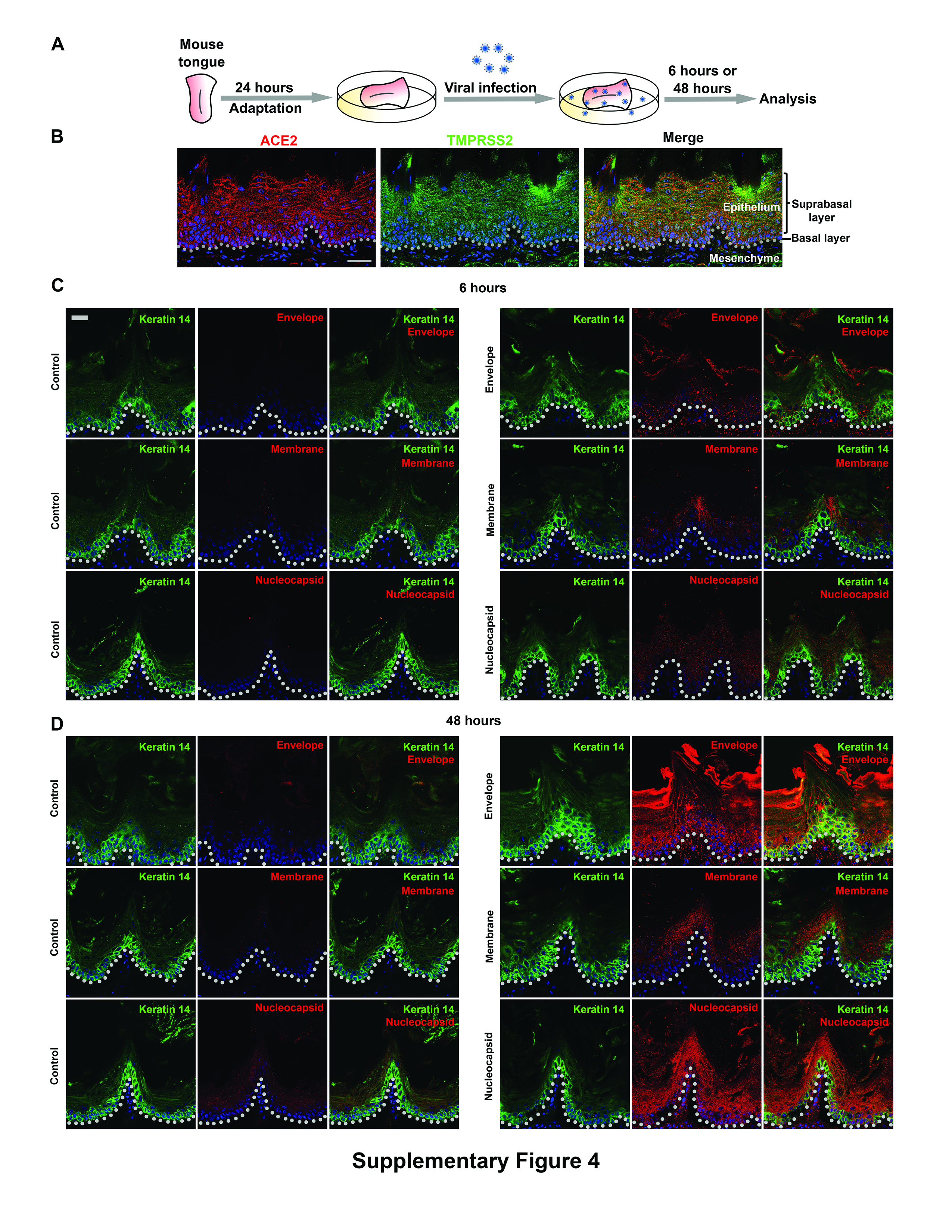

Supplement: Supplementary file 4 — Supplementary Figure 4 [file 41419_2026_8611_MOESM4_ESM.tif]

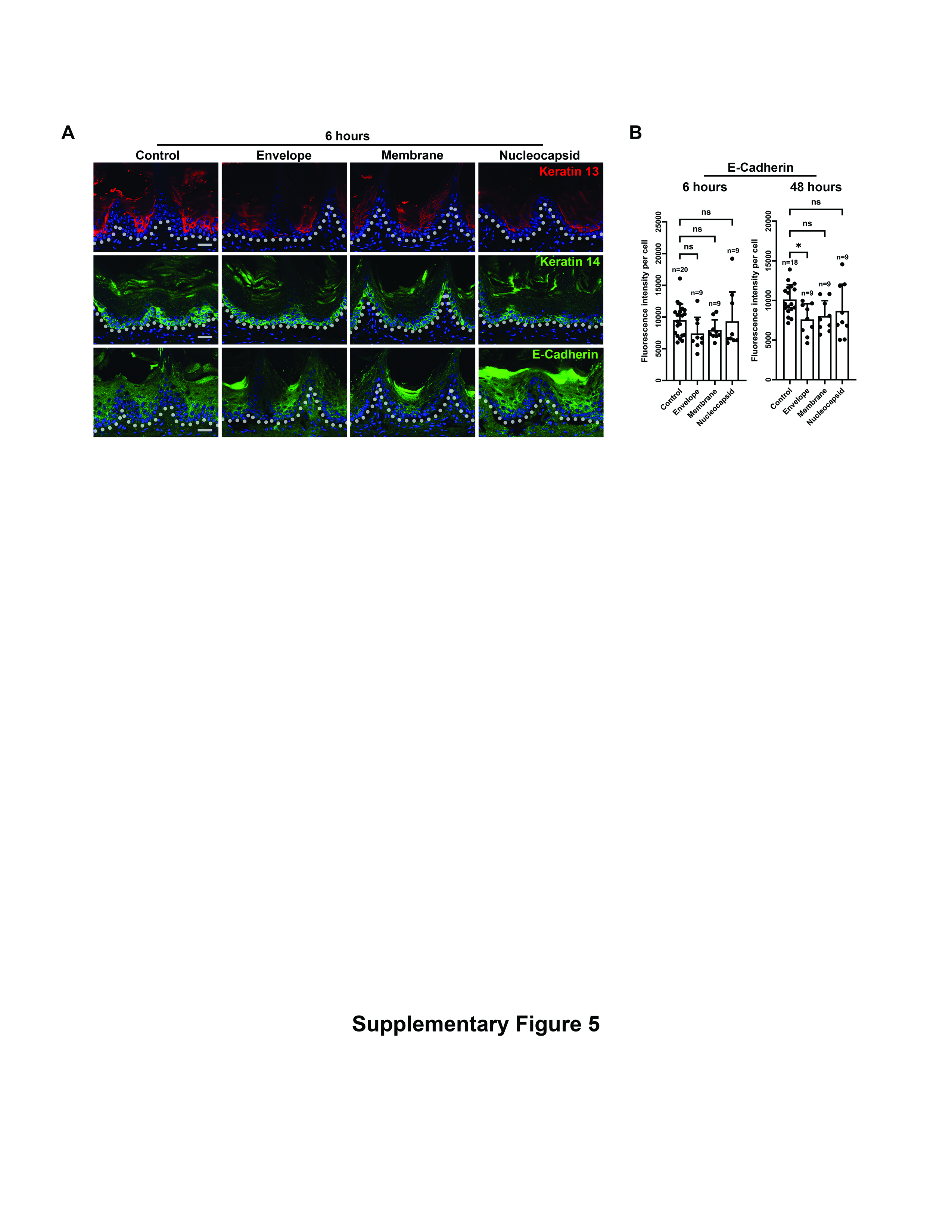

Supplement: Supplementary file 5 — Supplementary Figure 5 [file 41419_2026_8611_MOESM5_ESM.tif]

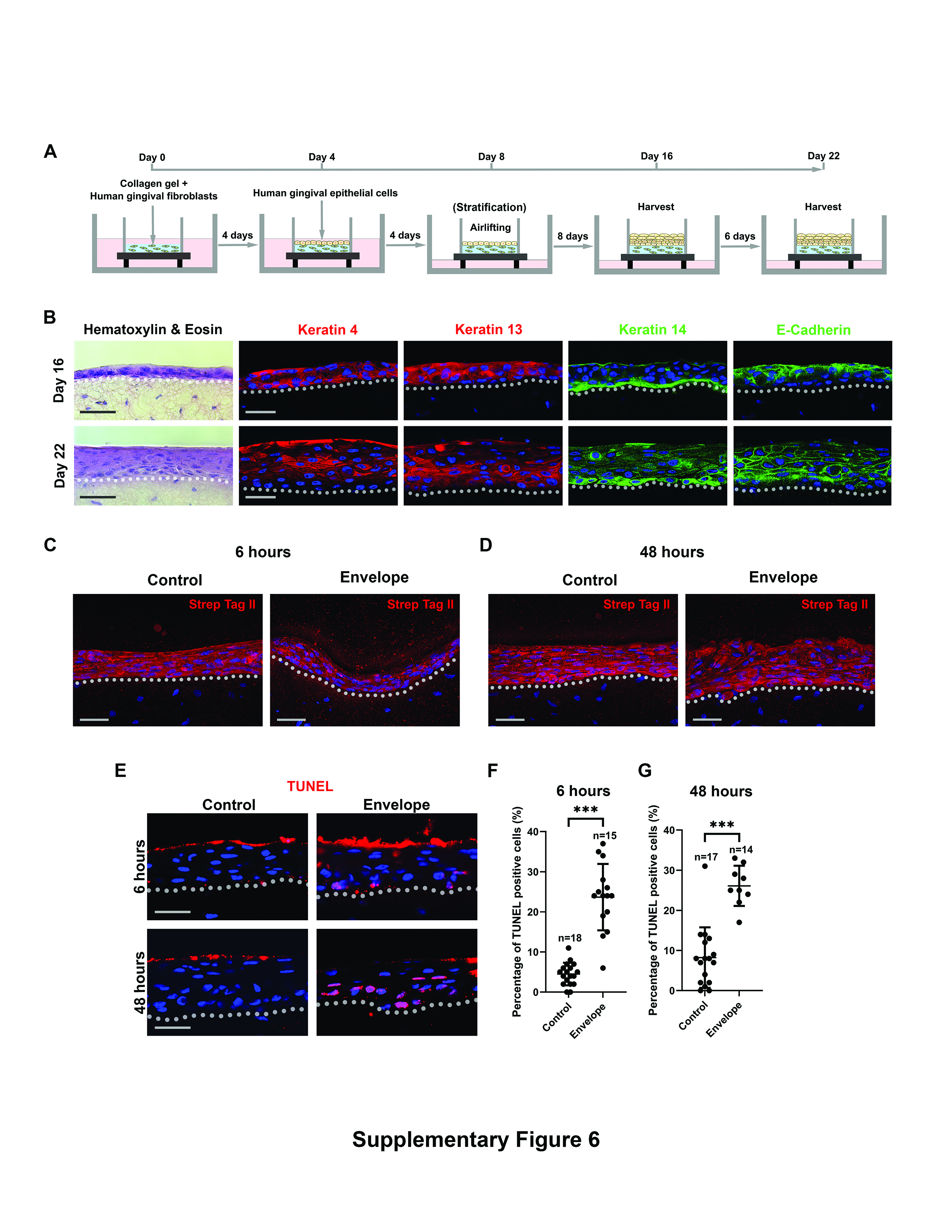

Supplement: Supplementary file 6 — Supplementary Figure 6 [file 41419_2026_8611_MOESM6_ESM.tif]

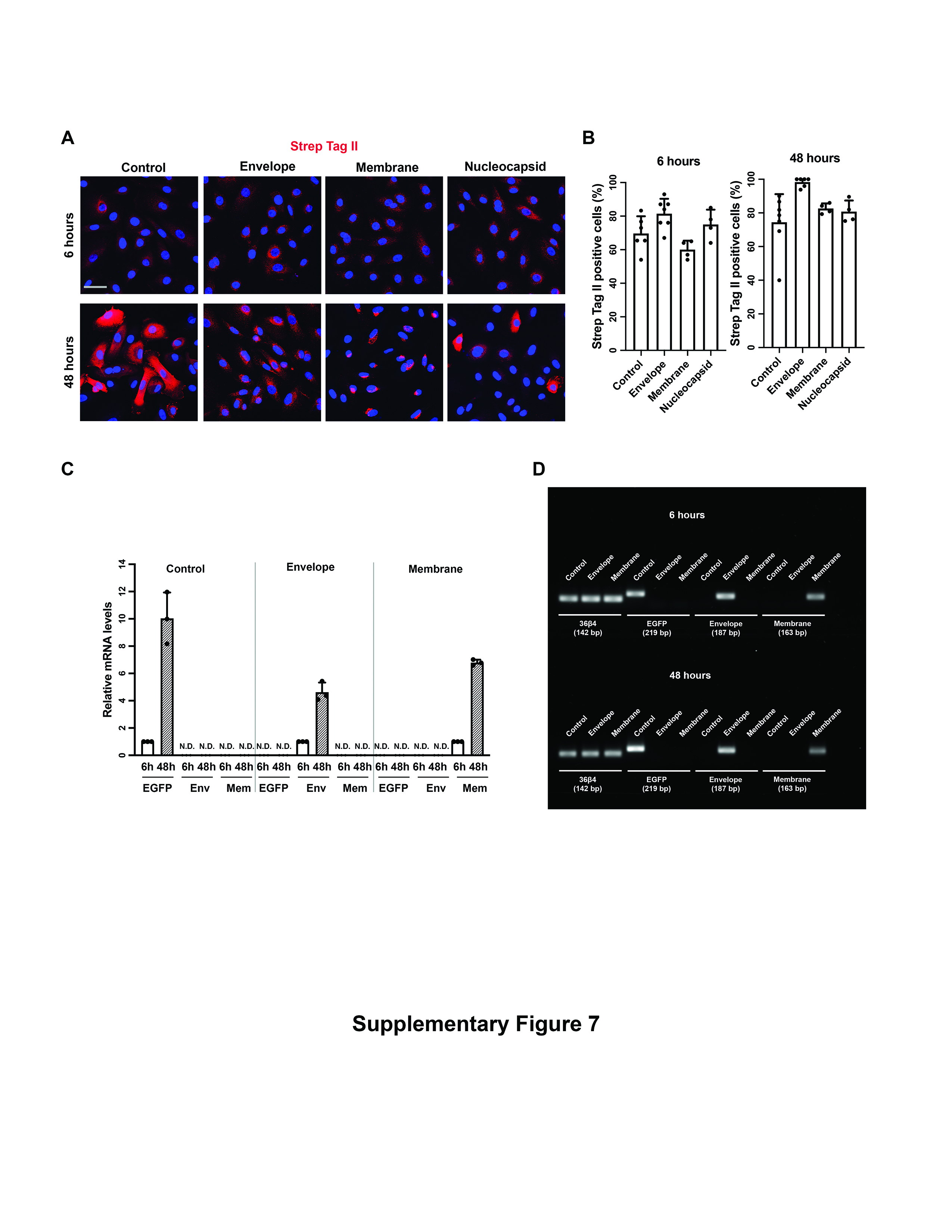

Supplement: Supplementary file 7 — Supplementary Figure 7 [file 41419_2026_8611_MOESM7_ESM.tif]

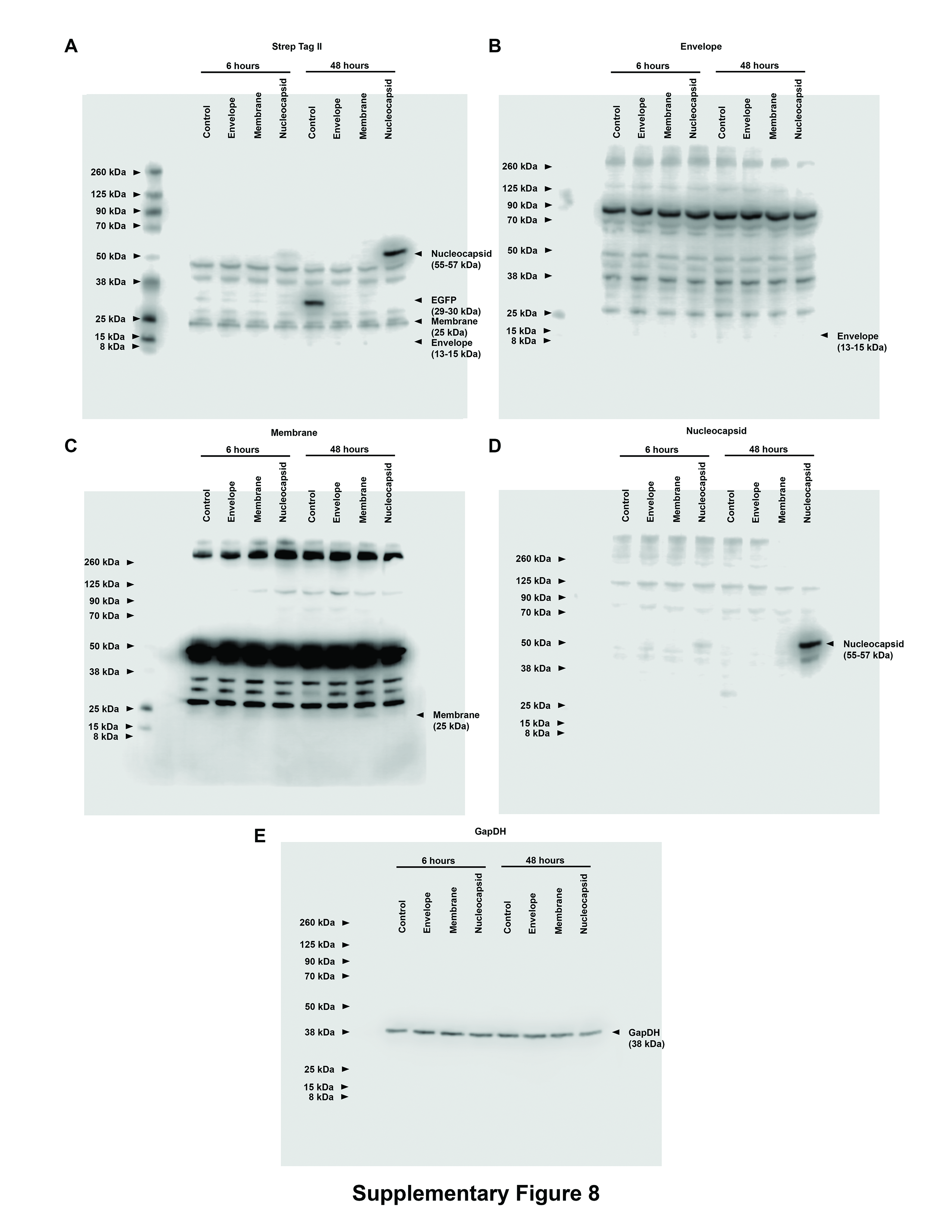

Supplement: Supplementary file 8 — Supplementary Figure 8 [file 41419_2026_8611_MOESM8_ESM.tif]

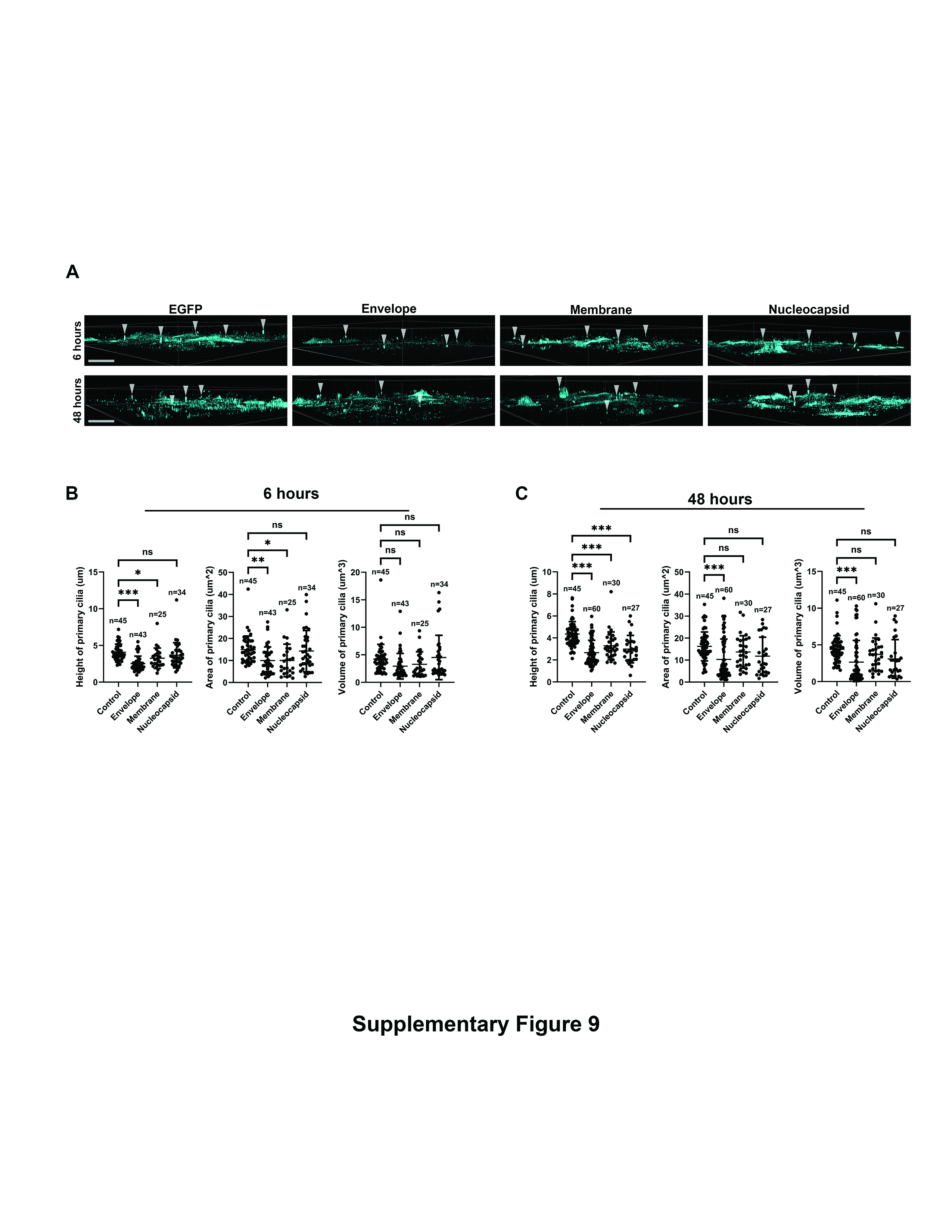

Supplement: Supplementary file 9 — Supplementary Figure 9 [file 41419_2026_8611_MOESM9_ESM.tif]

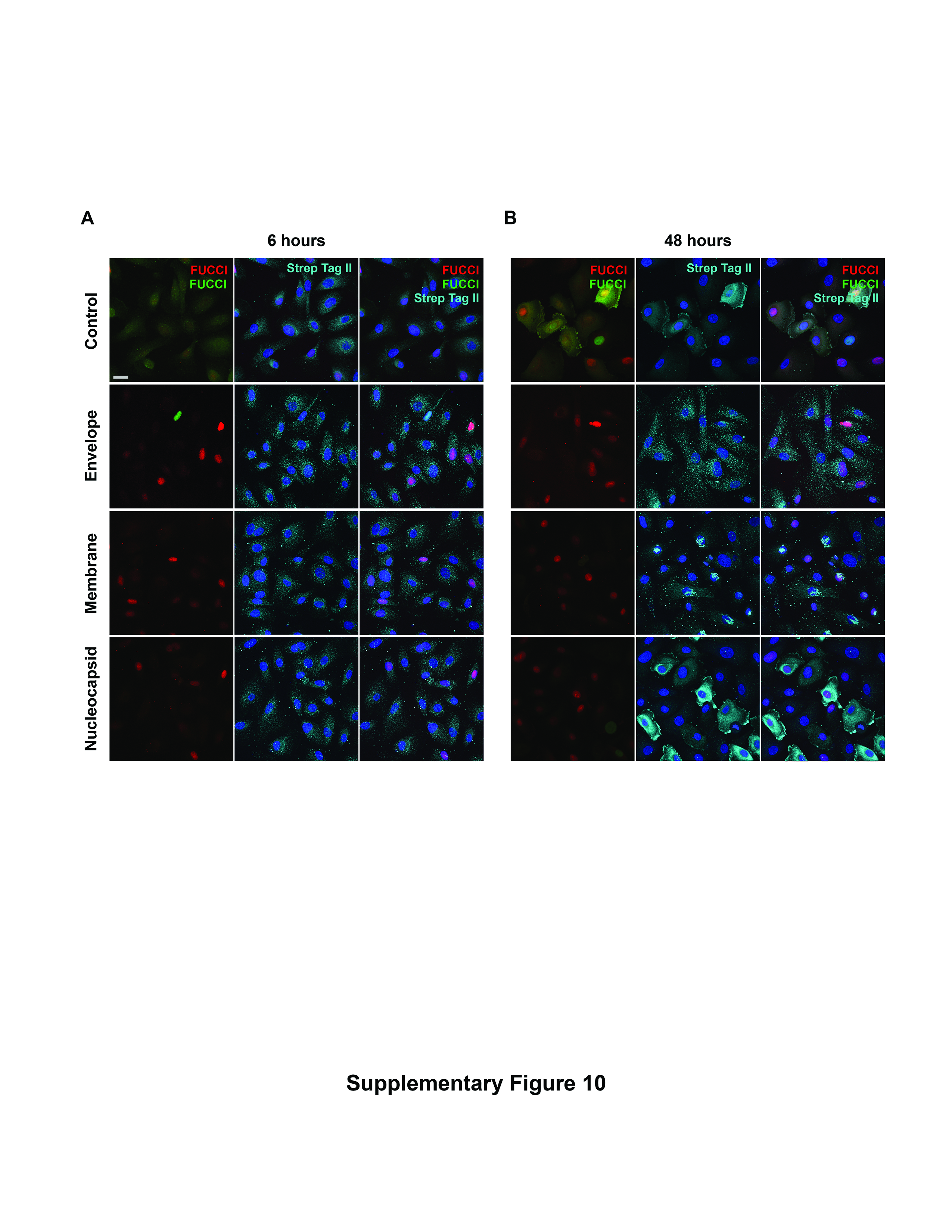

Supplement: Supplementary file 10 — Supplementary Figure 10 [file 41419_2026_8611_MOESM10_ESM.tif]

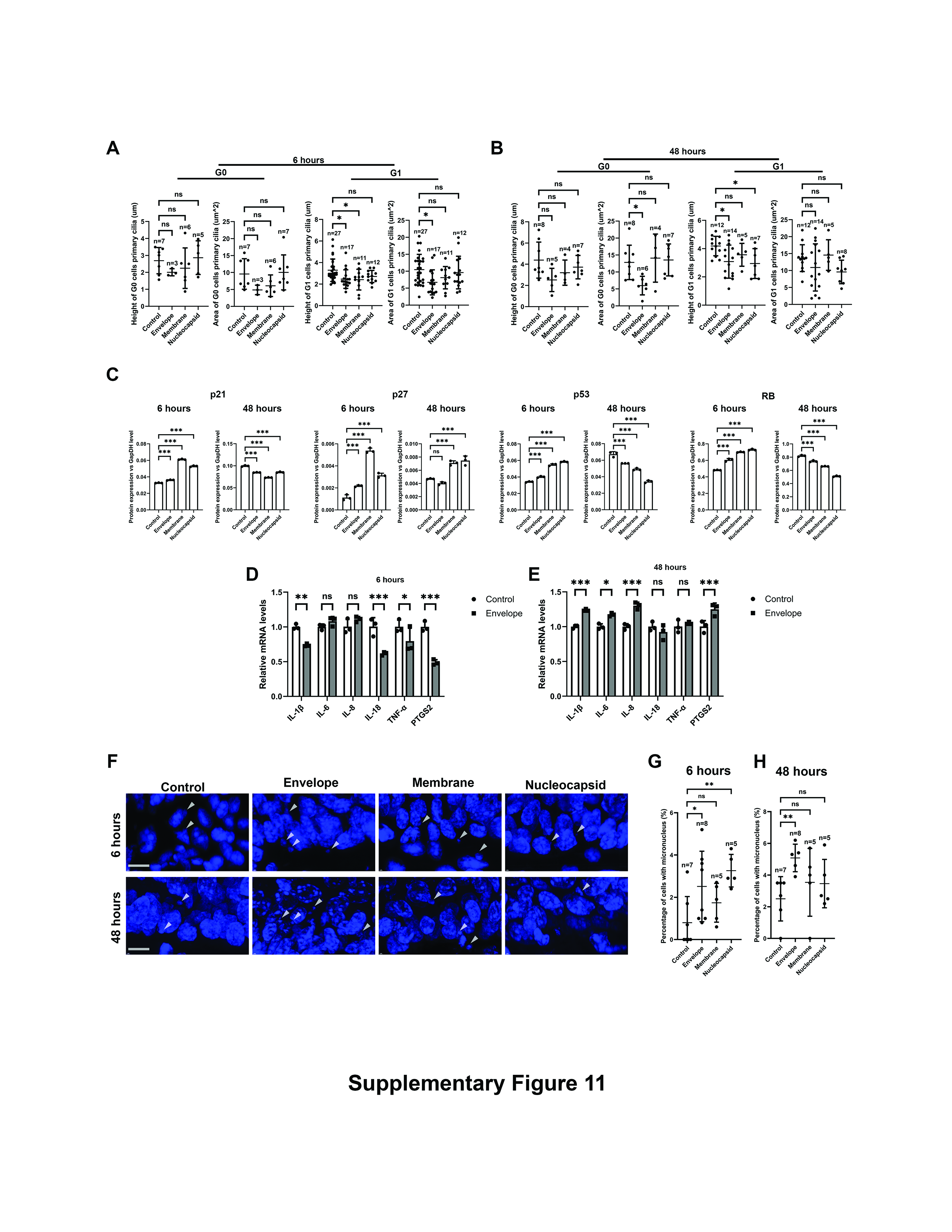

Supplement: Supplementary file 11 — Supplementary Figure 11 [file 41419_2026_8611_MOESM11_ESM.tif]

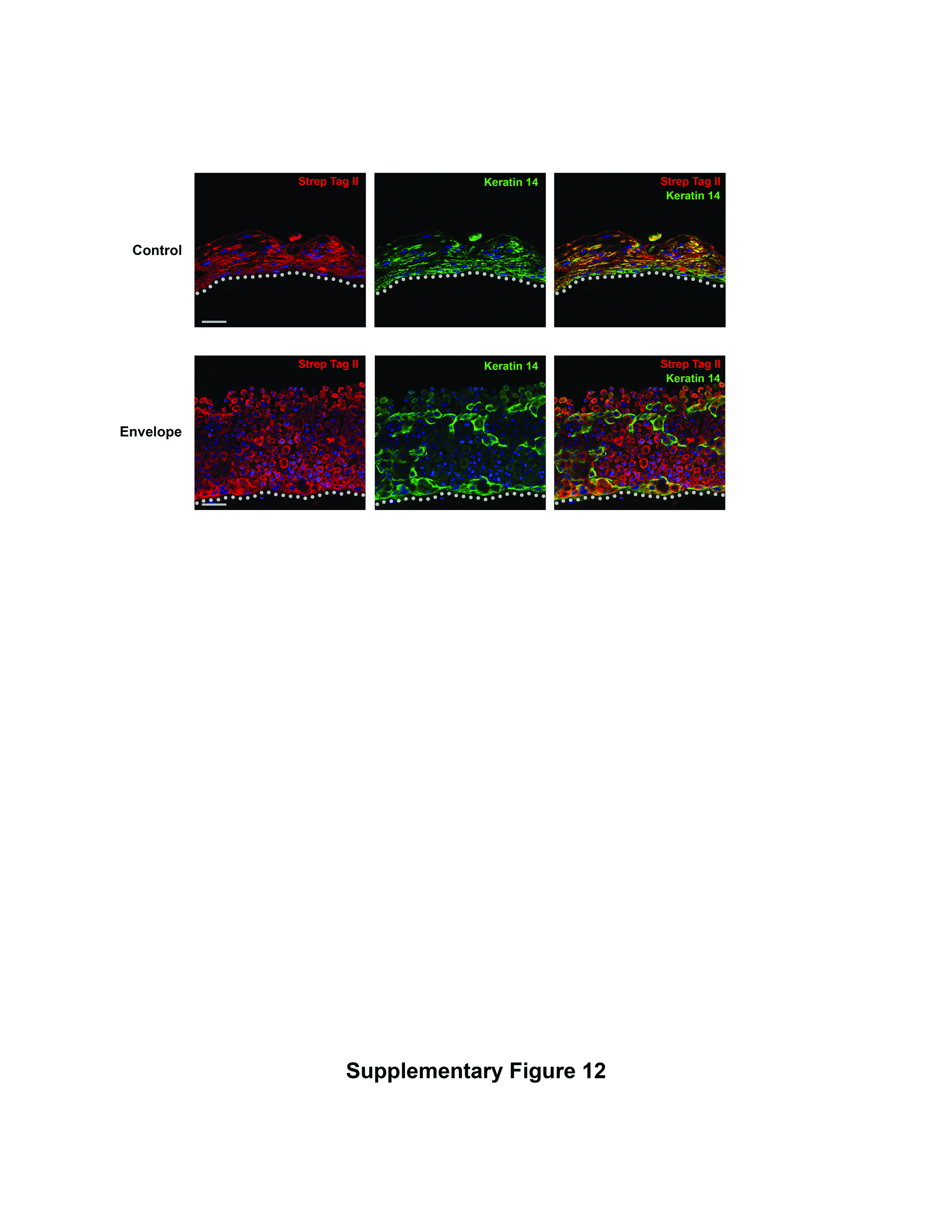

Supplement: Supplementary file 12 — Supplementary Figure 12 [file 41419_2026_8611_MOESM12_ESM.tif]

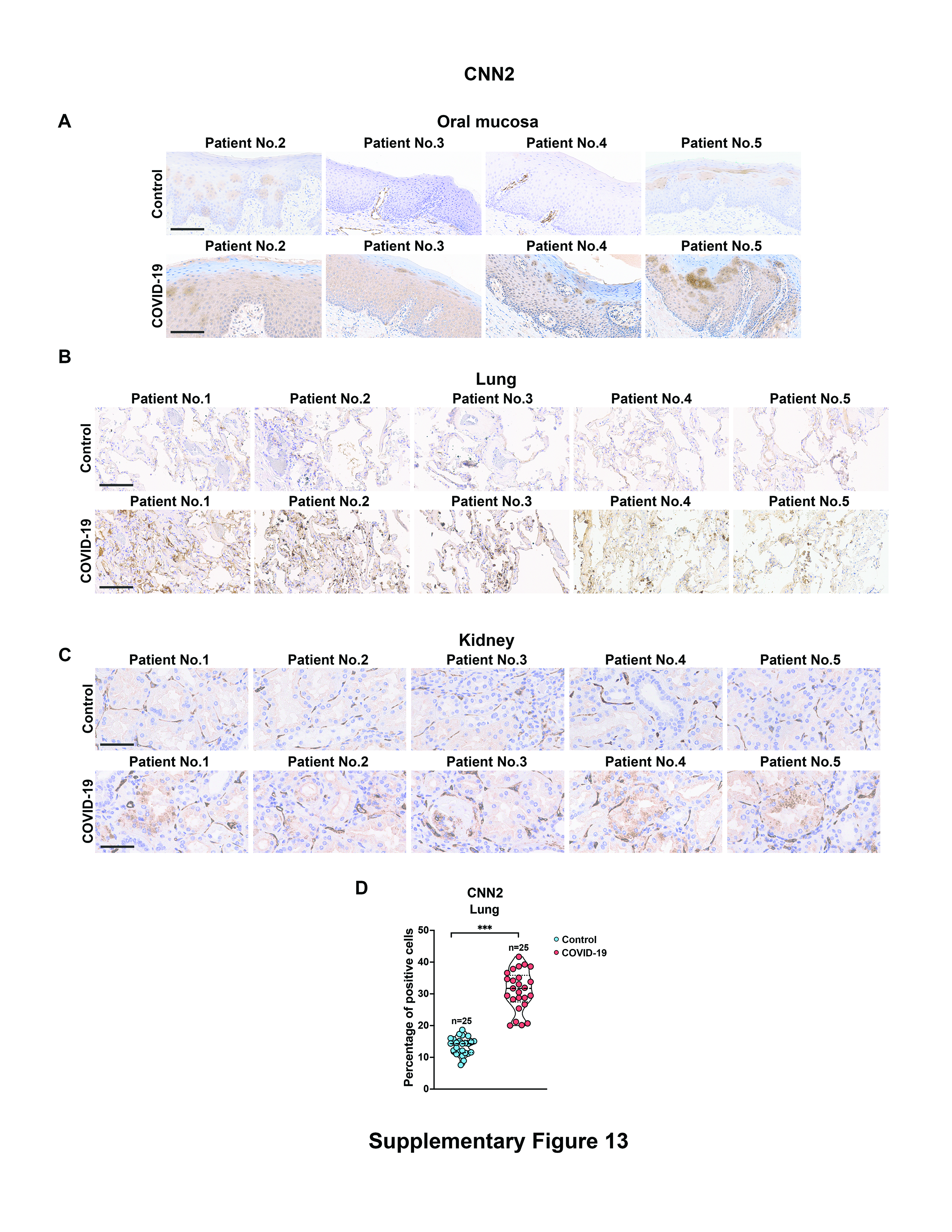

Supplement: Supplementary file 13 — Supplementary Figure 13 [file 41419_2026_8611_MOESM13_ESM.tif]

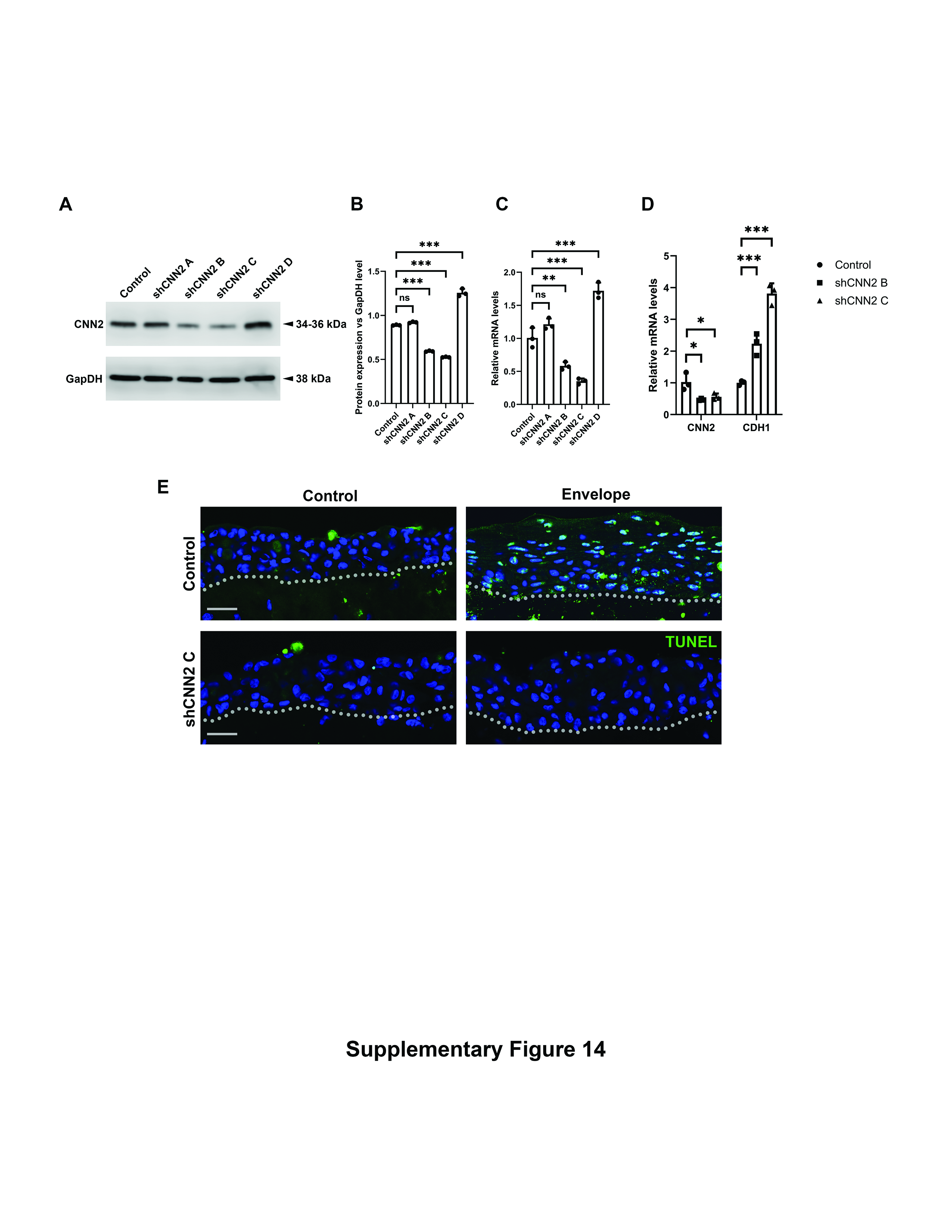

Supplement: Supplementary file 14 — Supplementary Figure 14 [file 41419_2026_8611_MOESM14_ESM.tif]

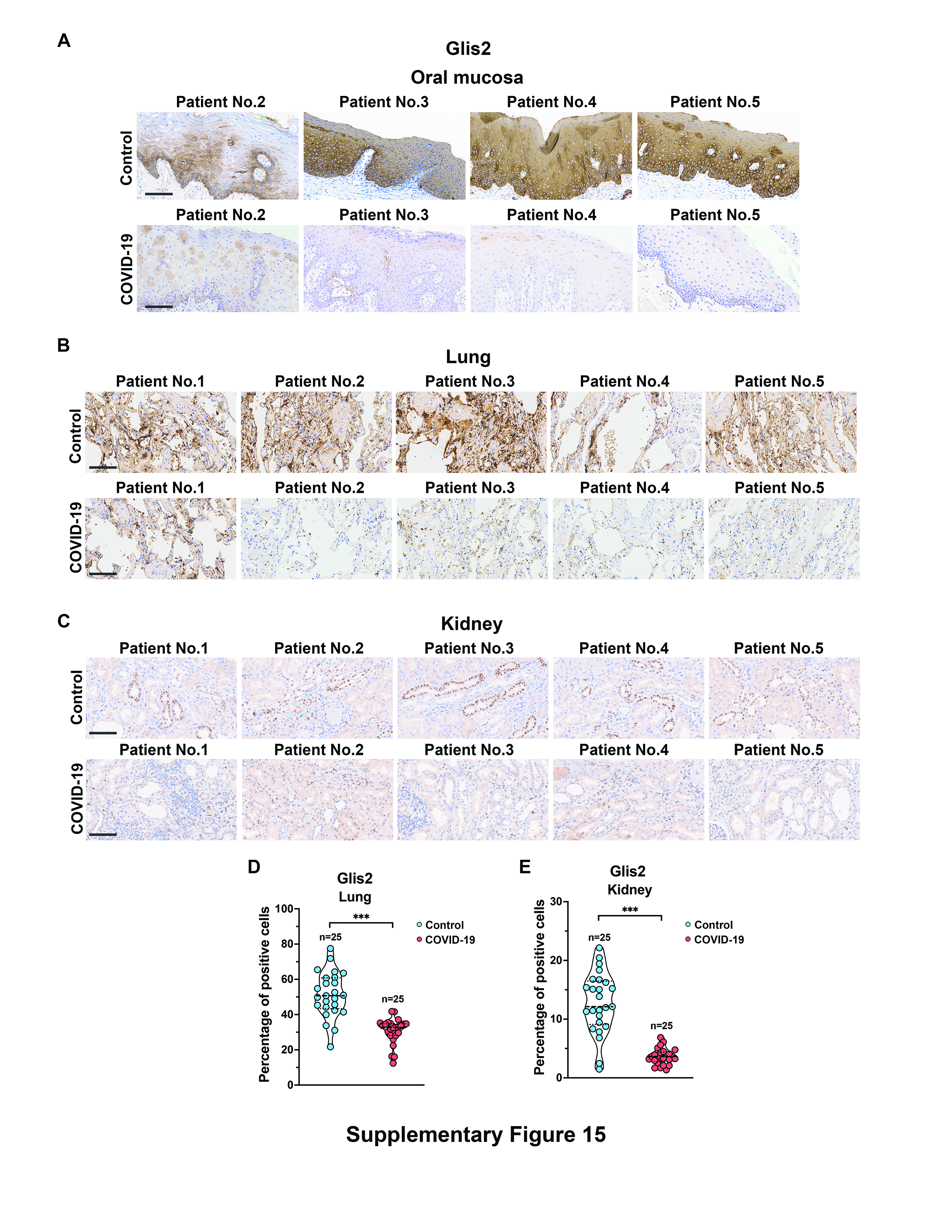

Supplement: Supplementary file 15 — Supplementary Figure 15 [file 41419_2026_8611_MOESM15_ESM.tif]

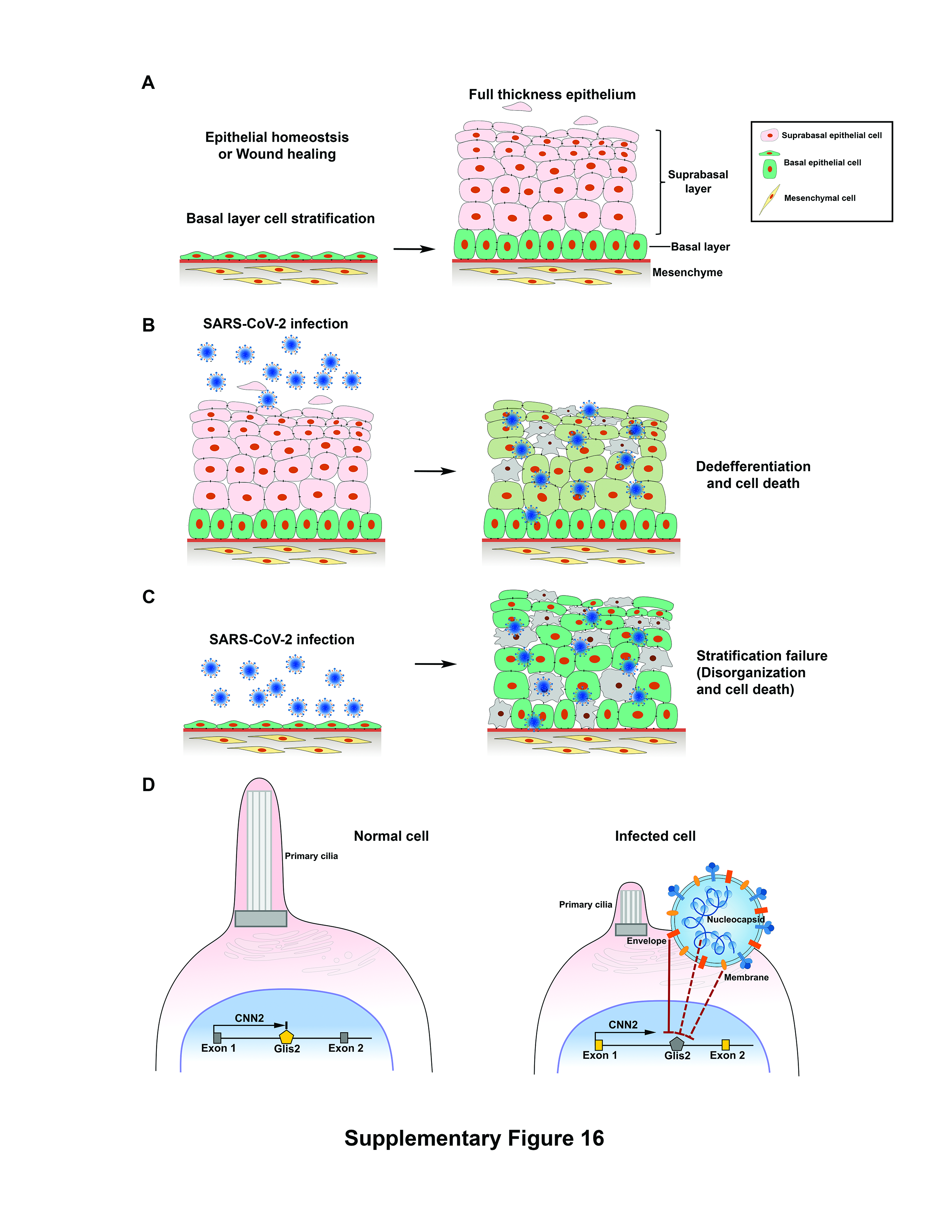

Supplement: Supplementary file 16 — Supplementary Figure 16 [file 41419_2026_8611_MOESM16_ESM.tif]

Figure 3M

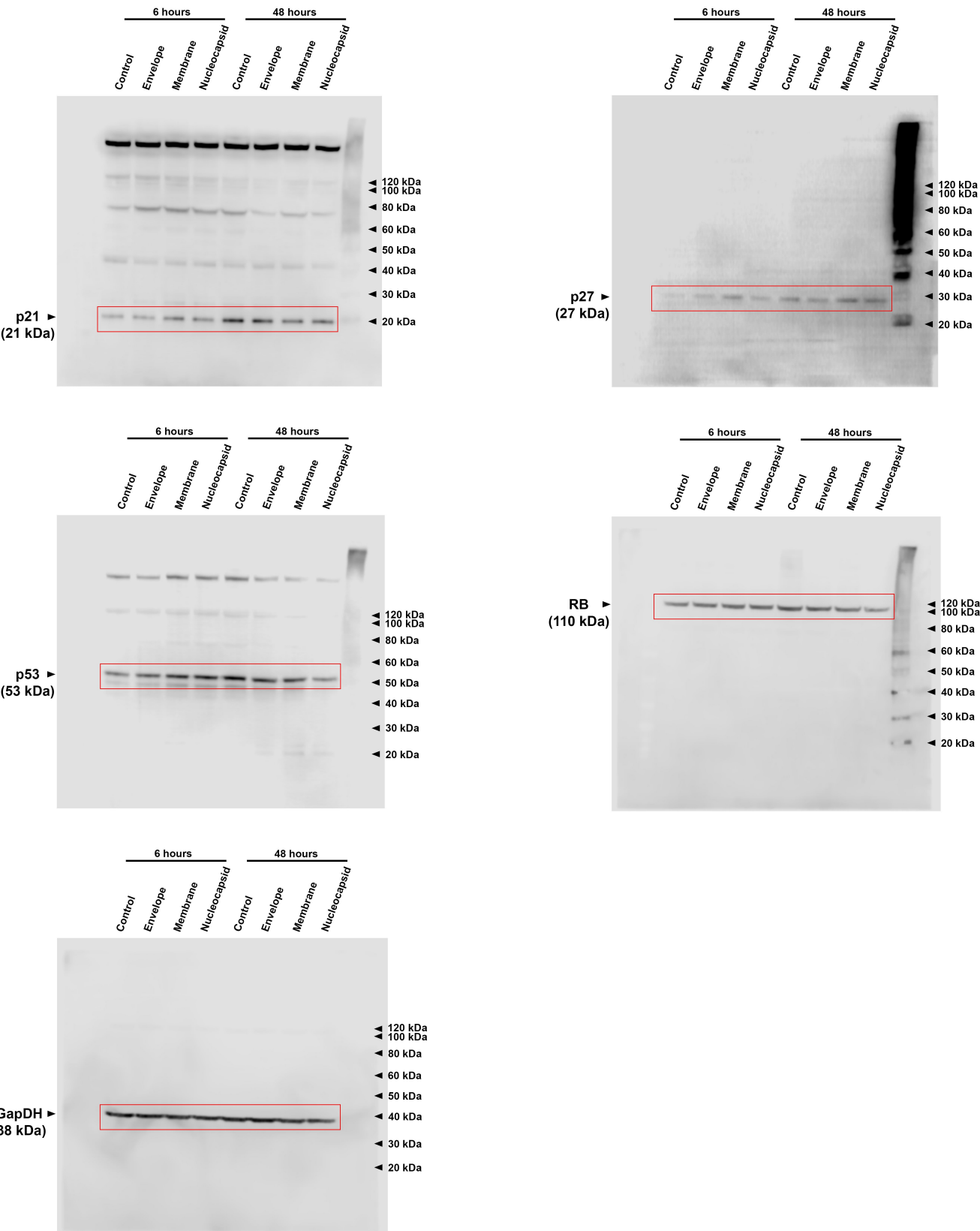

Figure 5C

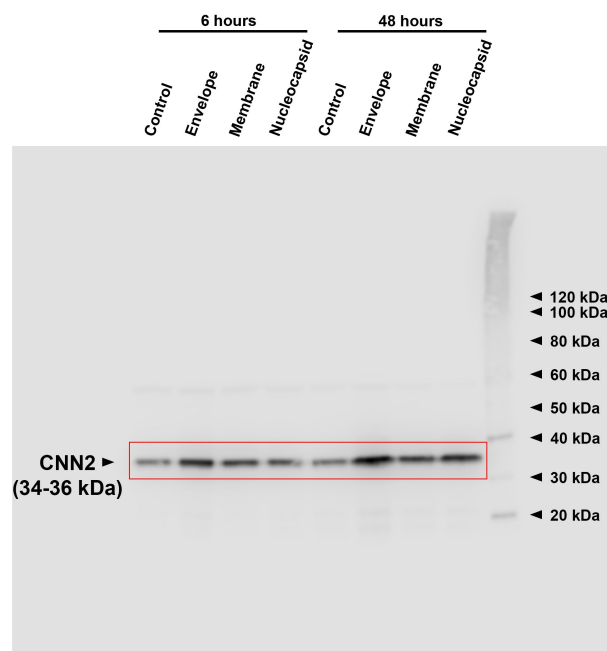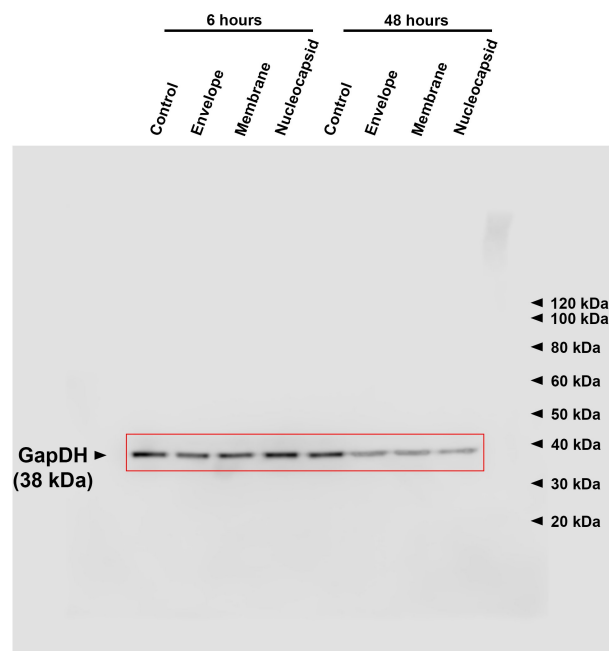

Figure 6K

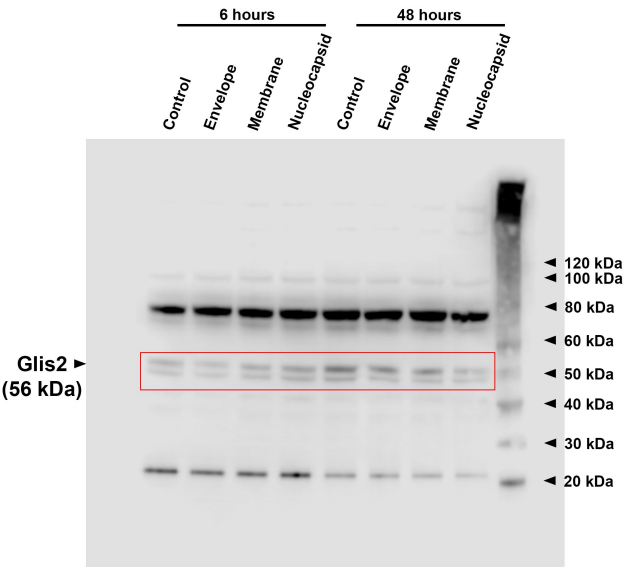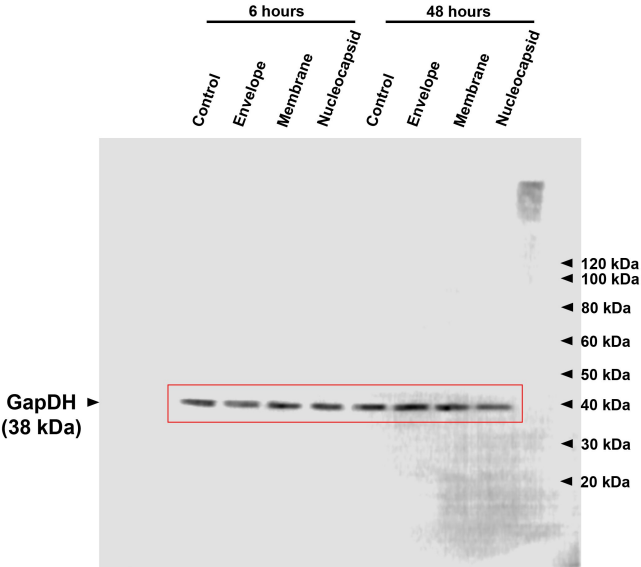

Supplementary Figure 14A

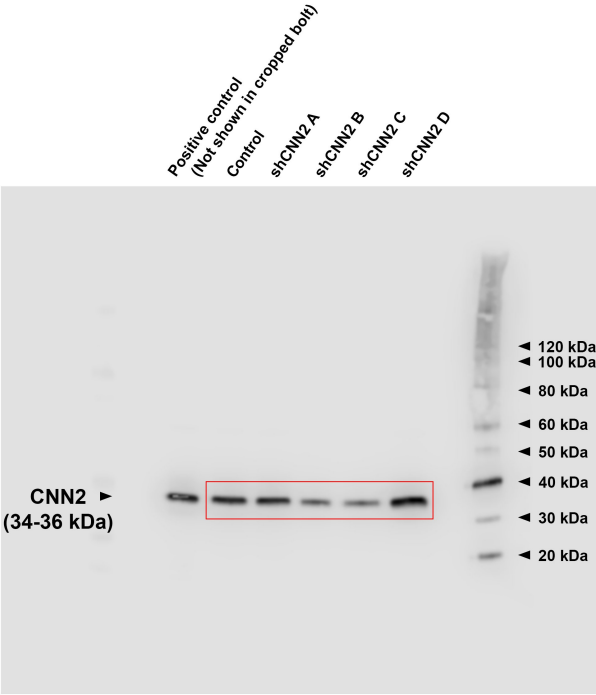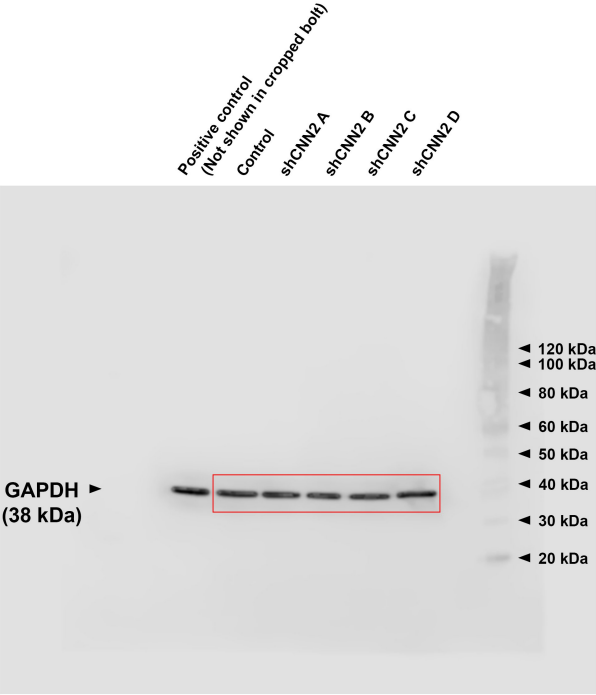

Supplement: Supplementary file 17 — Original data file for Western Blot [file 41419_2026_8611_MOESM17_ESM.pdf]
