# Supplementary material for: SARS-CoV-2 nonspike structural proteins hijack mucosa epithelial cell fate
Source: Cell Death Dis. 2026 Mar 23;17(1):340. doi: 10.1038/s41419-026-08611-6 (PMC13039937; doi:10.1038/s41419-026-08611-6)
Supplement: Supplementary file 18 — Supplementary Table 1 [file 41419_2026_8611_MOESM18_ESM.docx]

**Supplementary Table 1**

**Commonly upregulated molecules by Envelope protein (fold change>1.5)**

**at both time points**

| **Gene name** | **Protein name** | **6hrs** | **48hrs** |
| --- | --- | --- | --- |
| ALYREF | THO complex subunit 4 | 1.501688586 | 2.182666008 |
| ARPC4-TTLL3;ARPC4 | Actin-related protein 2/3 complex subunit 4 | 1.641892169 | 2.348036863 |
| CNN2 | Calponin;Calponin-2 | 3.143639208 | 5.723781845 |
| DYNC1H1 | Cytoplasmic dynein 1 heavy chain 1 | 1.800922622 | 2.745111732 |
| EEF1D | Elongation factor 1-delta | 2.124885103 | 2.143201745 |
| GSTP1 | Glutathione S-transferase P | 1.791114027 | 1.73399589 |
| IARS | Isoleucine--tRNA ligase, cytoplasmic | 1.829080095 | 1.862574956 |
| MARS | Methionine--tRNA ligase, cytoplasmic | 2.359876185 | 3.061153174 |
| MVP | Major vault protein | 1.766750879 | 7.993825154 |
| MYL6 | Myosin light polypeptide 6 | 1.7921101 | 2.706784358 |
| NME1-NME2;NME2;NME1 | Nucleoside diphosphate kinase;Nucleoside diphosphate kinase B | 1.885397909 | 1.645475133 |
| PDLIM4 | PDZ and LIM domain protein 4 | 1.943455138 | 1.843962178 |
| PFN1 | Profilin-1 | 1.528680985 | 1.586520947 |
| PPIA | Peptidyl-prolyl cis-trans isomerase A;Peptidyl-prolyl cis-trans isomerase A, N-terminally processed;Peptidyl-prolyl cis-trans isomerase | 1.630770375 | 1.556860007 |
| PSMD1 | 26S proteasome non-ATPase regulatory subunit 1 | 1.681351071 | 1.668307904 |
| RAI14 | Ankycorbin | 1.542911499 | 1.609242709 |
| RPL24 | 60S ribosomal protein L24 | 1.937839228 | 1.687167806 |
| RPL9 | 60S ribosomal protein L9 | 1.676999349 | 1.964407094 |
| RPS10;RPS10-NUDT3 | 40S ribosomal protein S10 | 1.595334269 | 2.051864162 |
| RPS12 | 40S ribosomal protein S12 | 1.612107795 | 1.555370164 |
| TMED10 | Transmembrane emp24 domain-containing protein 10 | 1.850374602 | 1.699437399 |
| VARS | Valine--tRNA ligase | 2.967748358 | 1.692443894 |

**Commonly upregulated molecules by Membrane protein (fold change>1.5)**

**at both time points**

| **Membrane group Gene names** | **Protein names** | **6hrs fold change** | **48hrs fold change** |
| --- | --- | --- | --- |
| ALDH1B1 | Aldehyde dehydrogenase X, mitochondrial | 1.572307596 | 2.186514074 |
| ALYREF | THO complex subunit 4 | 2.805249719 | 2.113791399 |
| ARPC4-TTLL3;ARPC4 | Actin-related protein 2/3 complex subunit 4 | 1.552827167 | 1.935442066 |
| CNN2 | Calponin;Calponin-2 | 2.212200209 | 2.220813752 |
| COPG1 | Coatomer subunit gamma-1 | 1.576167266 | 3.513995645 |
| FADS2 | Fatty acid desaturase 2 | 1.722515736 | 1.761848083 |
| LMAN1 | Protein ERGIC-53 | 1.687007374 | 2.266625377 |
| MRPL15 | 39S ribosomal protein L15, mitochondrial | 1.842423605 | 1.643373177 |
| MRPS34 | 28S ribosomal protein S34, mitochondrial | 1.772529521 | 1.722174936 |
| MYDGF | Myeloid-derived growth factor | 1.918070606 | 1.53954781 |
| NDUFV2 | NADH dehydrogenase [ubiquinone] flavoprotein 2, mitochondrial | 2.01956408 | 1.591168831 |
| NME1-NME2;NME2;NME1 | Nucleoside diphosphate kinase;Nucleoside diphosphate kinase B | 1.791329589 | 1.554762718 |
| PPIB | Peptidyl-prolyl cis-trans isomerase B | 1.662072128 | 1.977211988 |
| PRDX4 | Peroxiredoxin-4 | 1.601269217 | 1.790953253 |
| RPL24 | 60S ribosomal protein L24 | 2.565045992 | 1.504513625 |
| SRSF7 | Serine/arginine-rich splicing factor 7 | 2.014438096 | 1.591435672 |
| STX8 | Syntaxin-8 | 1.629316864 | 1.820339441 |
| TMED10 | Transmembrane emp24 domain-containing protein 10 | 1.674877378 | 1.693048536 |
| TMED7-TICAM2;TMED7 | Transmembrane emp24 domain-containing protein 7 | 1.992263104 | 2.289113125 |
| UBC;UBB;RPS27A;UBA52 | Polyubiquitin-C;Ubiquitin;Ubiquitin-40S ribosomal protein S27a;Ubiquitin;40S ribosomal protein S27a;Ubiquitin-60S ribosomal protein L40;Ubiquitin;60S ribosomal protein L40;Polyubiquitin-B;Ubiquitin | 1.599515049 | 1.657751362 |

**Commonly upregulated molecules by Nucleocapsid (fold change>1.5)**

**at both time points**

| **Gene name** | **Protein name** | **6hrs** | **48hrs** |
| --- | --- | --- | --- |
| CNN2 | Calponin;Calponin-2 | 1.626629301 | 3.623546871 |
| DYNC1H1 | Cytoplasmic dynein 1 heavy chain 1 | 2.210072463 | 1.737752471 |
| HDGF | Hepatoma-derived growth factor | 1.923468152 | 1.835968233 |
| NUMA1 | Nuclear mitotic apparatus protein 1 | 2.625046424 | 1.513922116 |
| RPS10;RPS10-NUDT3 | 40S ribosomal protein S10 | 1.57356086 | 1.856072246 |
| RPS17 | 40S ribosomal protein S17 | 1.627185575 | 2.082964476 |
| UGDH | UDP-glucose 6-dehydrogenase | 2.4368966 | 2.585606036 |

**Commonly upregulated molecules by Envelope protein (fold change<0.5)**

**at both time points**

| **Envelope group Gene names** | **Protein names** | **6hrs fold change** | **48hrs fold change** |
| --- | --- | --- | --- |
| ATP5F1 | ATP synthase F(0) complex subunit B1, mitochondrial | 0.131350151 | 0.480437766 |
| SLC25A1 | Tricarboxylate transport protein, mitochondrial | 0.302235846 | 0.378953903 |
| PSMA1 | Proteasome subunit alpha type-1 | 0.359760152 | 0.27574345 |
| PHB | Prohibitin | 0.487628086 | 0.496312449 |

**Commonly upregulated molecules by Membrane protein (fold change<0.5)**

**at both time points**

None

**Commonly upregulated molecules by Nucleocapsid protein (fold change<0.5)**

**at both time points**

None
