# Supplementary material for: SARS-CoV-2 nonspike structural proteins hijack mucosa epithelial cell fate
Source: Cell Death Dis. 2026 Mar 23;17(1):340. doi: 10.1038/s41419-026-08611-6 (PMC13039937; doi:10.1038/s41419-026-08611-6)
Supplement: Supplementary file 19 — Supplementary Table 2 [file 41419_2026_8611_MOESM19_ESM.docx]

**Supplementary Table 2**

**Oral mucosa**

| id_patient | age | sex | covid_test_type | location | diagnosis | group |
| --- | --- | --- | --- | --- | --- | --- |
| 1 | 44 | M | PCR | Palate | FH | Control |
| 2 | 38 | M | PCR | Palate | FH | Control |
| 3 | 15 | M | PCR | Palate | FH | Control |
| 4 | 20 | M | PCR | Palate | FH | Control |
| 5 | 16 | M | PCR | Palate | Normal | Control |
| 6 | 50 | M | PCR | Palate | FH | Control |
| 7 | 32 | F | PCR | Palate | FH | Control |
| 8 | 43 | M | PCR | Palate | FH | Control |
| 9 | 66 | M | PCR | Palate | Normal | Control |
| 10 | 28 | F | PCR | Tongue | FH | Control |
| 11 | 37 | M | PCR | Tongue | FH | Control |
| 12 | 57 | F | PCR | Tongue | FH | Control |
| 13 | 27 | F | PCR | Tongue | FH | Control |
| 14 | 18 | M | PCR | Tongue | FH | Control |
| 15 | 31 | M | PCR | Tongue | FH | Control |
| 16 | 23 | M | PCR | Lip | FH | Control |
| 17 | 67 | M | PCR | Lip | Chronic ulcer | Control |
| 18 | 60 | M | PCR | Lip | FH | Control |
| 19 | 50 | F | PCR | Lip | FH | Control |
| 20 | 64 | M | PCR | Gingival tissue | FH | Control |
| 21 | 60 | M | PCR | Gingival tissue | Gingivitis | Control |
| 22 | 25 | M | PCR | Gingival tissue | FH | Control |
| 23 | 65 | F | PCR | Gingival tissue | FH | Control |
| 24 | 45 | M | PCR | Gingival tissue | FH | Control |
| 25 | 60 | M | PCR | Gingival tissue | FH | Control |
| 26 | 65 | M | PCR | Gingival tissue | Normal | Control |
| 27 | 32 | F | PCR | Gingival tissue | FH | Control |
| 28 | 20 | M | PCR | Gingival tissue | FH | Control |
| 29 | 27 | F | PCR | Gingival tissue | FH | Control |
| 30 | 38 | F | PCR | Gingival tissue | FH | Control |
| 31 | 61 | M | PCR | Gingival tissue | Gingivitis | Control |
| 32 | 63 | F | PCR | Gingival tissue | FH | Control |
| 33 | 41 | M | PCR | Palate | Ulcerations | COVID |
| 34 | 58 | F | PCR | Palate | Vascular damage | COVID |
| 35 | 31 | F | PCR | Palate | Ulcerations | COVID |
| 36 | 76 | F | PCR | Palate | Ulcerations | COVID |
| 37 | 27 | M | PCR | Palate | Ulcerations | COVID |
| 38 | 88 | M | PCR | Palate | Ulcerations | COVID |
| 39 | 74 | F | PCR | Palate | Vascular damage | COVID |
| 40 | 35 | M | PCR | Palate | Ulcerations | COVID |
| 41 | 22 | M | PCR | Palate | Ulcerations | COVID |
| 42 | 77 | M | PCR | Tongue | Ulcerations | COVID |
| 43 | 69 | M | PCR | Tongue | Ulcerations | COVID |
| 44 | 71 | F | PCR | Tongue | Ulcerations | COVID |
| 45 | 74 | M | PCR | Tongue | Ulcerations | COVID |
| 46 | 27 | M | PCR | Tongue | Ulcerations | COVID |
| 47 | 46 | M | PCR | Tongue | Ulcerations | COVID |
| 48 | 30 | M | PCR | Lip | Ulcerations | COVID |
| 49 | 80 | M | PCR | Lip | Ulcerations | COVID |
| 50 | 64 | M | PCR | Lip | Ulcerations | COVID |
| 51 | 44 | F | PCR | Lip | Ulcerations | COVID |
| 52 | 26 | F | PCR | Gingival tissue | Ulcerations | COVID |
| 53 | 43 | F | PCR | Gingival tissue | Ulcerations | COVID |
| 54 | 44 | F | PCR | Gingival tissue | Ulcerations | COVID |
| 55 | 71 | M | PCR | Gingival tissue | Ulcerations | COVID |
| 56 | 65 | F | PCR | Gingival tissue | Ulcerations | COVID |
| 57 | 81 | M | PCR | Gingival tissue | Ulcerations | COVID |
| 58 | 54 | M | PCR | Gingival tissue | Ulcerations | COVID |
| 59 | 30 | M | PCR | Gingival tissue | Ulcerations | COVID |
| 60 | 70 | M | PCR | Gingival tissue | Ulcerations | COVID |
| 61 | 73 | M | PCR | Gingival tissue | Ulcerations | COVID |
| 62 | 60 | M | PCR | Gingival tissue | Ulcerations | COVID |
| 63 | 74 | F | PCR | Gingival tissue | Ulcerations | COVID |
| 64 | 55 | M | PCR | Gingival tissue | Ulcerations | COVID |

**Lung and Kidney**

| **id_patient** | **organ** | **group** | **age** | **sex** | **hypertension** | **diabetes** | **acute kidney lesion** |
| --- | --- | --- | --- | --- | --- | --- | --- |
| 1 | lung | control | 51 | M | No | No | No |
| 2 | lung | control | 42 | M | Yes | No | No |
| 3 | lung | control | 58 | M | No | Yes | No |
| 4 | lung | control | 55 | F | No | No | No |
| 5 | lung | control | 31 | F | Yes | Yes | No |
| 6 | lung | COVID | 62 | M | Yes | Yes | Yes |
| 7 | lung | COVID | 68 | F | Yes | No | Yes |
| 8 | lung | COVID | 71 | M | Yes | Yes | No |
| 9 | lung | COVID | 65 | F | No | Yes | Yes |
| 10 | lung | COVID | 51 | F | Yes | Yes | Yes |
| 11 | kidney | control | 23 | F | No | No | No |
| 12 | kidney | control | 48 | M | Yes | No | No |
| 13 | kidney | control | 53 | M | No | Yes | No |
| 14 | kidney | control | 61 | M | No | No | No |
| 15 | kidney | control | 72 | M | Yes | No | No |
| 16 | kidney | COVID | 78 | M | Yes | Yes | Yes |
| 17 | kidney | COVID | 69 | F | Yes | Yes | No |
| 18 | kidney | COVID | 55 | F | Yes | No | Yes |
| 19 | kidney | COVID | 62 | M | No | Yes | No |
| 20 | kidney | COVID | 75 | M | Yes | Yes | Yes |
