# Supplementary material for: SARS-CoV-2 nonspike structural proteins hijack mucosa epithelial cell fate
Source: Cell Death Dis. 2026 Mar 23;17(1):340. doi: 10.1038/s41419-026-08611-6 (PMC13039937; doi:10.1038/s41419-026-08611-6)
Supplement: Supplementary file 20 — Supplementary Table 3 [file 41419_2026_8611_MOESM20_ESM.docx]

**Supplementary Table 3**

The antibodies, primers, protein and viruses used in the study.

| **Primary antibodies (Immunofluorescence)** | | | | | | |
| --- | --- | --- | --- | --- | --- | --- |
| **Antigen** | **Figure** | **Final working concentration**  **(μg/ml)** | **Company** | **Cat. No.** | **Batch No.** |  |
| ACE2 | S4B | 5 | Biotechne | AF933 | HOK0620051 |  |
| Acetyl-Alpha Tubulin | 3A | 1 | Cell Signaling | 5335 | 4 |  |
| Acetylated tubulin | S9A | 0.5 | Sigma | T7451 |  |  |
| CNN2 | 5G,5I,5K | 2.5 | Proteintech | 21073-1-AP | 00014088 |  |
| E-Cadherin | 1C,2A,2E,4E,5G,5K,S5A,S6B | 2 | R&D | BAF748 |  |  |
| Glis2 | 6M | 5 | AVIVA | ARP30037_P050 | QC58201-161125 |  |
| Keratin 4 | 1A,2E,4B,5K,S6B | 3.7 | Abcam | ab51599 | GR149743-1 |  |
| Keratin 13 | 2A,S5A,S6B | 2 | Abcam | ab92551 | GR211864-2 |  |
| Keratin 14 | 1E,2A,2E,4B,4G,5K,S4C-D,S5A,S6B,S12 | 1 | Biolegend | 906004 | B365721 |  |
| SARS-CoV-2 Envelope | S4C,S4D | 0.2 | GeneTex | GTX136046 | 44377 |  |
| SARS-CoV-2 Membrane | S4C,S4D | 0.2 | GeneTex | GTX636246 | 45278 |  |
| SARS-CoV-2 Nucleocapsid | S4C,S4D | 0.2 | GeneTex | GTX135357 | 43979 |  |
| Strep Tag II | 3J,S6C-D,S7A,S10,S12 | 0.5 | Abcam | ab76949 | 1101072-14 |  |
| TMPRSS2 | S4B | 8.86 | Abcam | ab109131 | GR3343890-9 |  |

| **Secondary antibodies (Immunofluorescence)** | | | | |
| --- | --- | --- | --- | --- |
| **Target** | **Final working concentration**  **(μg/ml)** | **Company** | **Cat. No.** | **Batch No.** |
| Alexa 488 donkey anti-rat IgG | 6.67 | Life Technologies | A21208 | 1932496 |
| Alexa 488 donkey anti-rabbit IgG | 6.67 | Life Technologies | A21206 | 2072687 |
| Alexa 488 donkey anti-goat IgG | 6.67 | Life Technologies | A11055 | 2134018 |
| Alexa 488 goat anti-chicken IgY | 6.67 | Life Technologies | A11039 | 2420700 |
| Alexa 568 donkey anti-goat IgG | 4 | Life Technologies | A11057 | 2160061 |
| Alexa 568 donkey anti-mouse IgG | 4 | Life Technologies | A10037 | 1303018 |
| Alexa 568 donkey anti-rabbit IgG | 4 | Life Technologies | A10042 | 1964370 |
| Alexa 647 donkey anti-rabbit IgG | 4 | Life Technologies | A31573 | 2083195 |
| Alexa 647 donkey anti-mouse IgG | 4 | Life Technologies | A31571 | 2720365 |

| **Primary antibodies (Immunohistochemical Nanjing )** | | | | | | |
| --- | --- | --- | --- | --- | --- | --- |
| **Antigen** | **Figure** | **Final working concentration**  **(μg/ml)** | **Company** | **Cat. No.** | **Batch No.** |  |
| ACE2 | S1B | 5 | Biotechne | AF933 | HOK0620051 |  |
| TMPRSS2 | S1B | 8.86 | Abcam | ab109131 | GR3343890-9 |  |

| **Primary antibodies (Immunohistochemistry Brazil)** | | | | | | |
| --- | --- | --- | --- | --- | --- | --- |
| **Antigen** | **Figure** | **Final working concentration**  **(μg/ml)** | **Company** | **Cat. No.** | **Batch No.** |  |
| Cytokeratin 4 | 1A,S2A | 1.88 | Abcam | ab51599-1001 |  |  |
| Cytokeratin 14 | 1C,S2C | 1.88 | Abcam | ab119695 |  |  |
| Glis2 | 6I,S15A-C | 5 | AVIVA | ARP30037_P050 |  |  |
| CNN2 | 5E,S13A-C | 2.5 | Proteintech | 21073-1-AP |  |  |
| E-Cadherin | S2B | Prediluted | Dako | IR05961-2 |  |  |
| Cytokeratin 5/6 | 1G,S3A | Prediluted | Dako | IR78061-2 |  |  |
| Cytokeratin 8/18 | 1I,S3B | Prediluted | Dako | IR09461-2 |  |  |
| SARS-CoV-2 spike | S1C | 1:200 | BioSB | BSB-3701 |  |  |

| **Antibody (ChIP)** | | | | | |
| --- | --- | --- | --- | --- | --- |
| **Protein** | **Figure** | **Final working concentration**  **(μg/ml)** | **Company** | **Cat. No.** | **Batch No.** |
| Glis2 | 6D | 500 | AVIVA | ARP30037_P050 | QC58201-161125 |
| Rabbit IgG | 6D | 500 | Sigma | I5006-10MG | SLBW3651 |

| **Primary antibodies (Western Blotting)** | | | | | |
| --- | --- | --- | --- | --- | --- |
| **Protein** | **Figure** | **Final working concentration**  **(μg/ml)** | **Company** | **Cat. No.** | **Batch No.** |
| CNN2 | 5C,S8A | 0.5 | Proteintech | 21073-1-AP | 00014088 |
| GAPDH | 3M,5C,6K.S8E | 0.2 | Santa Cruz | SC-32233 | H2114 |
| Glis2 | 6K | 0.5 | AVIVA | ARP30037_P050 | QC58201-161125 |
| P21 | 3M | 0.9 | Proteintech | 10355-1-AP | 00145473 |
| P27 | 3M | 0.5 | Cell Signaling | 3686T |  |
| P53 | 3M | 0.3 | Proteintech | 10442-1-AP | 00107897 |
| RB | 3M | 0.5 | Cell Signaling | 9309T |  |
| SARS-CoV-2 Envelope | S8B | 0.1 | GeneTex | GTX136046 | 44377 |
| SARS-CoV-2 Membrane | S8C | 0.1 | GeneTex | GTX636246 | 45278 |
| SARS-CoV-2 Nucleocapsid | S8D | 0.1 | GeneTex | GTX135357 | 43979 |
| Strep Tag II | S8A | 0.1 | Abcam | ab76949 | 1101072-14 |

| **Secondary antibodies (Western Blotting)** | | | | |
| --- | --- | --- | --- | --- |
| **Target** | **Final working concentration**  **(μg/ml)** | **Company** | **Cat. No.** | **Batch No.** |
| HRP mouse | 0.184 | Cell Signalling | 7076 | 33 |
| HRP rabbit | 0.06 | Cell Signalling | 7074 | 28 |

| **Primers (human)** | | | |
| --- | --- | --- | --- |
| **Gene** | **Forward primer ('5-'3)** | **Reverse primer ('5-'3)** | **PCR**  **Product size** |
| 36β4 | GCAATGTTGCCAGTGTCTGT | GCCTTGACCTTTTCAGCAAG | 142 |
| CDH1 | AAGAAGGAGGCGGAGAAGAG | GGCTGTGGGGTCAGTATCAG | 203 |
| CNN2 | GTCCAAATATGACCCCCAGA | AACAGGTCCACAGGGTTCAT | 249 |
| GapDH | ATCACTGCCACCCAGAAGAC | CAGTGAGCTTCCCGTTCAG | 148 |
| Glis2 | AACGCCAGGTACAAGATGCT | GCTTAAAGCGGTCACTGGAG | 199 |
| IL-1β | GGAGAATGACCTGAGCACCT | GGAGGTGGAGAGCTTTCAGT | 185 |
| IL-6 | AGTCCTGATCCAGTTCCTGC | AAGCTGCGCAGAATGAGATG | 150 |
| IL-8 | TCCAAACCTTTCCACCCCAA | AAGCTGCGCAGAATGAGATG | 132 |
| IL-18 | GAGAAGTGTCCCAGGACATGA | CCCCCAATTCATCCTCTTTT | 128 |
| TNF-α | TGCTTGTTCCTCAGCCTCTT | GGTTTGCTACAACATGGGCTA | 199 |
| PTGS2 | ACCCTCTATCACTGGCATCCCCTTCT | TGCCTGCTCTGGTCAATGGAAGC | 224 |
| CDKN1a (P21) | CCCAAGCTCTACCTTCCCAC | ACAGGTCCACATGGTCTTCC | 121 |
| Keratin 13 | ACGCCAAGATGATTGGTT | AAGGCCTACGGACATCAGAA | 138 |
| Keratin 14 | CTCCTCCTCCCAGTTCTCCT | GGGATCTTCCAGTGGGATCT | 208 |
| EGFP | TATATCATGGCCGACAAGCA | GAACTCCAGCAGGACCATGT | 219 |
| SARS-CoV-2 Envelope | CCGGGACACTGATCGTAAAT | GGCACCCTGCTAGAATTGAG | 187 |
| SARS-CoV-2 Membrane | TATTCCTTTGGCTCCTGTGG | GTGCGTGCAAAAAGTCTGAA | 163 |
| CNN2_ ChIP_282bp | AGGTCAAGAACCGGGTGAGT | GATCTCAGACCTGGGTGCTC | 228 |
| CNN2_ ChIP_ neg ctrl _2 | GGCCTTTCCTTTCCTGTCTC | AGCCCCAGGTAGTGTTTCCT | 173 |

| **Primers (mouse)** | | | |
| --- | --- | --- | --- |
| **Gene** | **Forward primer ('5-'3)** | **Reverse primer ('5-'3)** | **PCR**  **Product size** |
| CNN2 | AGGAAGCAGAACTCCGAAGC | CCAGTTCTGCATAGAGCGGT | 158 |
| GapDH | ATCACTGCCACCCAGAAGAC | CAGTGAGCTTCCCGTTCAG | 148 |
| Glis2 | CAACGACCATCATGTCAAGC | CGGTTGTGGATCTTCAGGTT | 282 |
| CNN2_ ChIP_266bp | GGTCAAGAACCGGGTGAGT | CACAGCTTCCCACAGCAC | 166 |
| CNN2_ ChIP_ neg ctrl | AGGGGACCCTTGATCTCACT | TCTTAACATGCCCTGGCTCT | 208 |

| **Plasmids** | | |
| --- | --- | --- |
| **Vector** | **Company** | **Cat. No.** |
| pLVX-EF1alpha-eGFP-2xStrep-IRES-Puro | Addgene | 141395 |
| pLVX-EF1alpha-SARS-CoV-2-E-2xStrep-IRES-Puro | Addgene | 141385 |
| pLVX-EF1alpha-SARS-CoV-2-M-2xStrep-IRES-Puro | Addgene | 141386 |
| pLVX-EF1alpha-SARS-CoV-2-N-2xStrep-IRES-Puro | Addgene | 141391 |
| Ki67p-T2A-FUCCI | ^1^ |  |
| shCNN2 kit | Origene | TR305329 |
| shGlis2 | See Singer et al., ^1^ |  |

| **Cell apoptosis kit** | **Company** | **Cat. No.** |
| --- | --- | --- |
| In Situ Cell Death Detection Kit | Roche | Fluorescein, version 18 |
| In Situ Cell Death Detection Kit | Roche | TMR red, version 13 |

**Reference for Supplementary Table 3**

1 Singer, D. *et al.* Prominin-1 controls stem cell activation by orchestrating ciliary dynamics. *EMBO J* **38** (2019). <https://doi.org/10.15252/embj.201899845>
