# Supplementary material for: SARS-CoV-2 nonspike structural proteins hijack mucosa epithelial cell fate
Source: Cell Death Dis. 2026 Mar 23;17(1):340. doi: 10.1038/s41419-026-08611-6 (PMC13039937; doi:10.1038/s41419-026-08611-6)
Supplement: Supplementary file 21 — Supplementary Figures Caption [file 41419_2026_8611_MOESM21_ESM.docx]

**Fig. S1: Human samples analyzed in this study.**

**A,** Illustration of oral mucosa, lung and kidney epithelium used in this study.

**B,** Representative analysis of ACE2 and TMPRSS2 expression in human tongue epithelium.

**C,** Representative SARS-CoV-2 Spike protein staining in COVID-19 patient oral mucosa.

Bars: 100 μm.

**Fig. S2: Additional analysis of differentiation markers in oral mucosa of the control and COVID-19 patients.** Representative additional immunohistochemistry analysis images of the corresponding markers on COVID-19 patient samples.

Bars: 100 μm.

**Fig. S3: Additional analysis of differentiation markers in lung and kidney of the control and COVID-19 patients.** Representative additional immunohistochemistry analysis images of the corresponding markers on COVID-19 patient samples.

Bars: 100 μm.

**Fig. S4:** Ex vivo mouse tongue organ culture system.

**A,** Illustration of the ex vivo mouse tongue organ culture system.

**B,** Representative images for ACE2 and TMPRSS2 expression in mouse tongue epithelium.

**C, D,** Immunostaining of the mouse tongue organ cultures used in the study with E, M, N specific antibodies. Note the infection spanned into the full layers of the epidermis already at 6 hours and more significantly at 48 hours.

Bars: B: 20 μm; C, D: 10 μm

**Fig. S5: Ex vivo mouse tongue organ culture shows Keratins and E-Cadherin changes in the mouse oral epithelium upon SARS-CoV-2 structural protein infection.**

**A, B,** Additional data for Figs. 2A-C, with indicated markers in the corresponding samples. Dotted lines indicate epithelial-mesenchymal junctions. n = number of the image fields quantified.

For statistical test results, center values represent mean and error bars represent s.d.. ns: no significance (p>=0.05); * p<0.05

Bars: 20 μm.

**Fig. S6: Full thickness 3D human oral mucosa system.**

**A,** Illustration of the system.

**B,** Histology and immunofluorescence analysis of the differentiation markers at two indicated stages.

**C, D,** Strep Tag II staining on the control and E protein infected full thickness 3D oral mucosa equivalent.

**E-G,** TUNEL and quantification analysis on the full thickness 3D system. Dotted lines indicate epithelial-mesenchymal junctions.

For statistical test results, center values represent mean and error bars represent s.d.. n = number of the image fields quantified. ns: no significance (p>=0.05); * p<0.05; ** p<0.01; *** p<0.001. Bars: 20 μm.

**Fig. S7: Validation of SARS-CoV-2 non-spike structural proteins infection efficiency in the HGEPp cells.**

**A, B,** Immunofluorescent staining of Strep Tag II protein using specific antibodies on the cultured cells. The efficiency was quantified for positive cell percentages (B). Each dot in (B) represent one random field that contains around 50 cells.

**C, D,** Real time RT-PCR analysis of the control (EGFP), E, M proteins’ mRNA expression levels at 6 hours and 48 hours after infection (note the highly increased expression at 48 hours). The PCR products were loaded on gel to verify the specificity.

For statistical test results, center values represent mean and error bars represent s.d.. N.D: not detected.

Bar: 20 μm.

**Fig. S8: Western Blotting validation of SARS-CoV-2 non-spike structural proteins infection efficiency in the HGEPp cells.**

**A-E**, Western blotting was performed on the indicated samples, with specific antibodies. Please note while the control (EGFP) and N protein could be readily visualized especially at 48 hours. The E and M protein could only be detected weakly, suggesting the technique is not optimal for validating SARS-CoV-2 structural protein presence in the cells unless the levels are very significant.

**Fig. S9: Additional data for SARS-CoV-2 non-spike structural proteins can cause cilia changes.**

**A,** Representative images for analyzing cilia in the growing HGEPp cells under indicated conditions. White arrowheads indicate cilia. **B, C,** Quantification of cilia size in the indicated conditions in HGEPp cells. n = the number of cilia quantified.

For statistical test results, center values represent mean and error bars represent s.d.. * p<0.05; ** p<0.01; *** p<0.001.

Bars: A: 20 μm.

**Fig. S10: Validation of SARS-CoV-2 structural protein infection in the FUCCI system.**

**A, B,** HGEPp cells carrying FUCCI system were infected with Control, E, M and N sequence containing virus, then counterstained with Strep Tag II antibodies. Note the signals were stronger in the 48 hours samples.

Bars: 10 μm.

**Fig. S11: Additional data for SARS-CoV-2 non-spike structural proteins cause cellular and cell cycle changes.**

**A, B,** Additional analysis of cilia size at indicated time points and conditions. n = the number of cilia quantified. **C,** Quantification of western blotting showed in Fig. 3M. **D, E,** Real time RT-PCR analysis of cytokine and PTGS2 expression in HGEPp cells infected with control or Envelope protein. **F-H,** Micronuclei analysis in culture mouse tongue epitheliums. White arrowheads indicate micronuclei. n = number of the image fields quantified.

For statistical test results, center values represent mean and error bars represent s.d.. ns: no significance (p>=0.05); * p<0.05; ** p<0.01; *** p<0.001.

Bars: F: 10 μm.

**Fig. S12: Validation of SARS-CoV-2 E protein infection in the 3D oral epithelium stratification model.**

The human oral mucosa 3D stratification system (Fig.2D, Fig.4A-G, Fig. 5I, J) were stained with Strep Tag II and K14 antibodies. Note that the Strep Tag II signal spans the full thickness of the epidermis and not restricted to K14 positive or negative cells.

Bars: 20 μm.

**Fig. S13: Additional analysis of CNN2 in oral mucosa, Lung and Kidney samples of the control and COVID-19 patients.** **A-C,** Representative additional immunohistochemistry analysis images for CNN2 on COVID-19 patient samples (please note CNN2 expression increased in the kidney duct epithelial cells). **D,** Quantification for lung samples. n = number of the image fields quantified.

For statistical test results, center values represent mean and error bars represent s.d..*** p<0.001.

Bars: 100 μm.

**Fig. S14: Additional data for shCNN2 analysis. A, B,** Western blotting validation of a group of shCNN constructs infected in HGEPp cells. Notes shCNN2 No.B and C were used for the study. **C,** Real time RT-PCR validation of CNN2 expression in the shRNA infected cells. **D,** CNN2 and CDH1 (E-Cadherin) expression in shCNN2 treated cells. **E,** TUNEL analysis on control or Envelope treated control or shCNN2 cells. Dotted lines indicate epithelial-mesenchymal junctions.

For statistical test results, center values represent mean and error bars represent s.d.. ns: no significance (p>=0.05); * p<0.05; ** p<0.01; *** p<0.001.

Bars: 20 μm

**Fig. S15: Additional analysis of Glis2 in oral mucosa, Lung and Kidney samples of the control and COVID-19 patients.** **A-C,** Representative additional immunohistochemistry analysis images for Glis2 on COVID-19 patient samples. **D, E,** Quantification for lung and kidney samples. n = number of the image fields quantified.

For statistical test results, center values represent mean and error bars represent s.d.. *** p<0.001.

Bars: 100 μm.

**Fig. S16: Schematic summary of the findings of the study. A,** Basal layer cells of the epithelium are responsible for replenishing normal epidermis or wound healing. **B,** SARS-CoV-2 infection on full thickness epidermis cause dedifferentiation. **C,** SARS-CoV-2 infection on basal cells only cause epithelial stratification failure. **D,** The cellular and molecular changes caused by SARS-CoV-2 nonspike proteins are connected with Glis2 controlled CNN2 expression.
